# The *tarani* mutation alters surface curvature in *Arabidopsis* leaves by perturbing the patterns of surface expansion and cell division

**DOI:** 10.1093/jxb/erv015

**Published:** 2015-02-24

**Authors:** Premananda Karidas, Krishna Reddy Challa, Utpal Nath

**Affiliations:** Department of Microbiology and Cell Biology, Indian Institute of Science, Bangalore 560 012, India

**Keywords:** *Arabidopsis thaliana*, cell proliferation, gibberellic acid (GA), leaf shape, surface curvature, *TARANI*.

## Abstract

The leaf surface usually stays flat, maintained by coordinated growth. Growth perturbation can introduce overall surface curvature, which can be negative, giving a saddle-shaped leaf, or positive, giving a cup-like leaf. Little is known about the molecular mechanisms that underlie leaf flatness, primarily because only a few mutants with altered surface curvature have been isolated and studied. Characterization of mutants of the *CINCINNATA*-like *TCP* genes in *Antirrhinum* and *Arabidopsis* have revealed that their products help maintain flatness by balancing the pattern of cell proliferation and surface expansion between the margin and the central zone during leaf morphogenesis. On the other hand, deletion of two homologous *PEAPOD* genes causes cup-shaped leaves in *Arabidopsis* due to excess division of dispersed meristemoid cells. Here, we report the isolation and characterization of an *Arabidopsis* mutant, *tarani* (*tni*), with enlarged, cup-shaped leaves. Morphometric analyses showed that the positive curvature of the *tni* leaf is linked to excess growth at the centre compared to the margin. By monitoring the dynamic pattern of *CYCLIN D3;2* expression, we show that the shape of the primary arrest front is strongly convex in growing *tni* leaves, leading to excess mitotic expansion synchronized with excess cell proliferation at the centre. Reduction of cell proliferation and of endogenous gibberellic acid levels rescued the *tni* phenotype. Genetic interactions demonstrated that *TNI* maintains leaf flatness independent of *TCP*s and *PEAPOD*s.

## Highlight

Isolation and characterization of a novel *Arabidopsis* mutant is reported. This has a cup-shaped leaf surface, as opposed to the flat leaves of wild-type plants.

## Introduction

The leaf surface is typically flat and is thought to be under genetic control. An early and regulated growth pattern (Kuchen *et al.*, 2012; Remmler and Rolland-Lagan, 2012) is essential for maintaining flatness, and its perturbation can lead to altered surface architecture (Todd, 1985; Coen *et al.*, 2004). For example, if the margin grows more relative to the centre, the leaf surface is expected to buckle to form a saddle-like shape with negative Gaussian curvature (Prusinkiewicz and Barbier de Reuille, 2010), which can be measured as a product of the two principal linear curvatures (Kreyszig, 1991; Nath *et al.*, 2003). Conversely, if the centre grows more relative to the margin, the lamina would form a cup shape with positive curvature. Control of surface curvature has been studied mostly from a biomechanical perspective (Moulia, 2000; Sharon *et al.*, 2002; Klein *et al.*, 2007; Koehl *et al.*, 2008; Liang and Mahadevan, 2009) and little is known about its genetic regulation in leaves, primarily due to the lack of mutants with altered surface curvature, and because the analysis of surface curvature in an expanding organ is difficult (Moulia, 2000; Sharon *et al.*, 2002).

Leaf growth commences with the initiation of the primordium and continues until the leaf reaches its maximum size. An early pattern of growth, which is maintained throughout the growth phase, is established at the primordial stage with maximum growth rate at the base and progressively less towards the tip (Kuchen *et al.*, 2012). Growth is primarily a result of two cellular processes: division and expansion. Based on the mitotic status of the cells, expansion can be of two types: proliferation associated or mitotic expansion and differentiation associated. A newly divided leaf pavement cell measures up to ~100 μm^2^ in area and expands to approximately double its volume before the next division. This mitotic expansion is characteristically different from differentiation-associated expansion, which takes place when the cell exits the mitotic cycle and enters into terminal differentiation. At this stage, a pavement cell can expand up to ~60 times in area by cell wall loosening and usually does not display mitotic activity under normal circumstances (Donnelly *et al.*, 1999), even though more recent studies predict division of mature pavement cells (Asl *et al.*, 2011). Thus, differentiation-associated expansion plays an important role in leaf growth, whereas proliferation, along with mitotic expansion, provides the organ with more cells.

Leaf kinematic analysis shows that mitotic expansion and differentiation-associated expansion in *Arabidopsis* leaves are temporally and spatially separated, though with some overlap. Early leaf growth is predominantly contributed by cell division and mitotic expansion, while the later part of growth is primarily due to cell expansion coupled with terminal differentiation (Beemster *et al.*, 2005; Asl *et al.*, 2011). Cell maturity and enlargement are first initiated at the tip of a young leaf and progressively spread towards the base during leaf morphogenesis (Avery, 1933; Donnelly *et al.*, 1999; Nath *et al.*, 2003). Thus, a growing leaf is constituted of larger, differentiated cells at the tip and smaller, proliferating cells at the base, while the interfacing transition region contains cells with both characters. Since increasing number of cells exit the mitotic cycle and enter differentiation, it gives the transition zone an appearance of a moving mitotic arrest front from tip to base, described as the primary arrest front (Nath *et al.*, 2003; White, 2006). A more recent analysis of leaf growth suggests that the size of the proliferative zone, which remains anchored at the lamina–petiole junction, is relatively constant throughout leaf growth, before disappearing rather abruptly (Andriankaja *et al.*, 2012). A second mitotic arrest of the dispersed meristematic cells (DMCs), which give rise to specialized cell types such as stomata and vascular cells, has been proposed (White, 2006). Similar to the primary arrest, DMC arrest is initiated at the tip and seems to progress towards the base as the leaf expands and matures. The DMC arrest is, however, slower than the primary arrest front and is regulated by an independent genetic mechanism.

A large number of genes affecting leaf growth have been isolated and their mutants studied. There are groups of genes that regulate either cell proliferation and mitotic expansion or differentiation-associated expansion (Tsuge *et al.*, 1996; Kim *et al.*, 2003). Among the genes affecting proliferation, there are negative as well as positive regulators. The promoters of cell proliferation include *GROWTH*-*REGULATING FACTOR*s (*GRF*s) and *GRF*-*INTERACTING FACTORS* (*GIF*s), *AINTEGUMENTA* (*ANT*), *NAC1*/*ATAF2*, *ARGOS*, the vacuolar H^+^-pyrophophatase *AVP1*, *JAGGED*, *KLUH*, and *UBP15* (Mizukami and Fischer, 2000; Horiguchi *et al.*, 2005; Li *et al.*, 2005; Anastasiou *et al.*, 2007; Liu *et al.*, 2008; Gonzalez *et al.*, 2012). Overexpression of these genes increases organ size because of either longer duration or faster rate of cell proliferation and mitotic expansion, whereas loss of function in several of them causes smaller organs made of fewer cells. Some of the factors that suppress cell proliferation are *ARF2*, *PEAPOD* (*PPD*), *TCP*, *DA1*/*DAR*, *BIG BROTHER*, and *ABAP1* (Nath *et al.*, 2003; Palatnik *et al.*, 2003; Okushima *et al.*, 2005; Disch *et al.*, 2006; Schruff *et al.*, 2006; White, 2006; Efroni *et al.*, 2008; Li *et al.*, 2008; Masuda *et al.*, 2008). Loss of function of these genes causes increased organ size and more cell division, and their overexpression can reduce cell number and, consequently, organ size. Compensation between cell division and cell size also plays an important role in controlling organ size (Day and Lawrence, 2000). According to this phenomenon, a decrease in cell number is often partly compensated by a concomitant increase in cell size, and vice versa (Horiguchi *et al.*, 2005; Ferjani *et al.*, 2007).

Even though organ size is altered when expression of these genes is perturbed, leaf flatness is seldom affected. Indeed, mutations in only two groups of homologous genes, *CINCINNATA*-like *TCP*s and *PEAPOD*s (*PPD*s), are reported to influence curvature of the lamina. Mutations in both these genes cause excess cell proliferation and mitotic expansion, leading to larger leaf size, while their gain of function results in smaller leaves composed of fewer cells (Nath *et al.*, 2003; Palatnik *et al.*, 2003; White, 2006; Efroni *et al.*, 2008; Koyama *et al.*, 2010; Sarvepalli and Nath, 2011). However, there are marked differences between the ways these two genes inhibit cell proliferation and mitotic expansion. *CIN*-like *TCP* genes regulate the primary arrest of cell division and suppress proliferation-related mitotic expansion more at the margin than in the centre. Consequently, loss of *CIN* function results in preferential cell proliferation and surface expansion in the margin leading to negative surface curvature. By contrast, *PPD* genes regulate the arrest of the mitotic growth of the DMCs, and their loss of function results in downward cup-shaped leaves with positive surface curvature.

Studies on genetic control of leaf flatness have been impeded by the limited number of mutants isolated with altered curvature, and because dissection of their geometrical, kinematical, and developmental phenotype is difficult. Consequently, the cellular basis of surface curvature and a genetic framework that regulates this phenomenon has not yet emerged. Here we address this issue by isolating and characterizing a new *Arabidopsis* mutant, *tarani* (*tni*), that produces leaves with upwardly curved, coracle-shaped laminae and by establishing its genetic interactions with other curvature-forming mutants.

## Materials and methods

### Plant materials and growth conditions

The *Arabidopsis thaliana* (L.) Heynh. ecotypes Col-0 and L*er* were used as wild-type controls. The mutant lines *tcp4-2* (GABI_363408), *tcp10-2* (SALK_050423), *arf2-8* (CS24602), and *Δppd* (CS16548) were obtained from Stock Centre (http://arabidopsis.org/). The *tcp2-1* (SAIL 562-D05) line was a kind gift from Pilar Cubas, Spain. The *jaw-D*, *35S::ICK2*, *proCyclin D3;2::GUS*, *klu-4*, and *ga1-3* lines have been previously reported (De Veylder *et al.*, 2001; Palatnik *et al.*, 2003; Tyler *et al.*, 2004; Anastasiou *et al.*, 2007; Dewitte *et al.*, 2007). The *tni jaw-D*, *tni jaw-D CyclinD3;2::GUS*, *tni tcp2 tcp4 tcp10*, *Δppd jaw-D*, and *Δppd tcp4 tcp10* lines were genotyped and selected in the F_3_ generation (list of primers used is given in Supplementary Table S1). EMS-mutagenesis was carried out on Col-0 seeds as described previously (Kim *et al.*, 2006) and the mutants were screened in the F_2_ generation.

Seeds were sown on plates containing Murashige Skoog medium (catalogue number PT011-1L; Himedia, India) with or without antibiotic, stratified for 3 days in the dark, and moved to a growth room. Seedlings with the first pair of true leaves were transplanted onto soil (Keltech Co., Bengaluru, India). All experiments were performed with plants grown under long-day conditions (16h light/8h dark) at 22°C in a custom-made walk-in Plant Growth Room (Research and Test Equipment Co., Bengaluru, India).

### Mapping of *tni*

The *tni* mutant in the Col-0 background was crossed with L*er* to generate a mapping population, and mapping was carried out as described previously (Jander *et al.*, 2002; Weigel and Glazebrook, 2002). The mutation was mapped to an interval between markers NGA162 and CIW11 on the third chromosome. New CAPS markers TN3C6.4, TN3C6.7, TN3C7.09, and TN3C7.5 were made and the interval was narrowed down to ~0.4Mb, between the TNI3C7.09 and TNI3C7.5 markers (primer sequences are given in Supplementary Table S1). A total of 418 mutant plants were screened. The 0.4Mb interval contained 120 annotated genes.

### Leaf measurements

Fifth leaves >4mm long were sandwiched between glass slides and photographed using a Canon Power-shot S90 camera. For leaves <4mm long, photographs were taken after sandwiching on a Wild Heerberg trinocular microscope fitted with a Nikon Coolpix 4500 camera. Cup-shaped leaves of *tni* and *ppd* mutants were flattened after introducing incisions at the margin, and care was taken not to include the newly exposed edges while measuring the perimeter (White, 2006). Shape parameters of the crinkly leaves of *jaw-D* were measured as described previously for *cin* leaves (Nath *et al.*, 2003). Briefly, crinkly *jaw-D* leaves were cut into several pieces and each piece was completely spread into a plane between glass slides. Leaf margin was measured for individual pieces, excluding the cut edges, and summed to obtain the perimeter of the whole leaf. In our growth conditions, mature fifth leaves of Col-0, *tni*, and *ppd* did not show any serration. Minor serrations observed for *jaw-D* leaves were ignored and measurement was performed through the middle of the serrations. Measurements of length, width, area, and perimeter were made in the photographs of the flattened leaves or leaf pieces using the straight and free-hand lines tool of the Image J software (*rsbweb.nih.gov/ij/*). Average ± SEM values are presented in Table 1. The significance of differences was determined by Student’s *t*-test and the *P* values are listed in Supplementary Table S2.

**Table 1.** Shape and size parameters of the mature fifth leaves of wild-type (Col-0) and mutant plants

Genotype	Length (mm)	Width (mm)	Perimeter (mm)	Area (mm^2^)	Length:width ratio	SEM	Perimeter/√Area	SEM	Sample size
Col-0	12.8±0.8	9.0±0.4	36.3±1.6	90.3±6.8	1.4	0.04	3.8	0.01	10
*tni*	14.2±1.5	16.2±1.4	42.7±3.7	179.5±28.4	0.9	0.02	3.2	0.06	9
*jaw-D*	14.7±1.8	10.3±1.0	47.6±4.4	118.6±17.2	1.4	0.05	4.4	0.10	9
*tni jaw-D*	15.2±1.8	15.1±2.1	87.4±16.9	267±57.8	1.0	0.03	5.3	0.19	8
L*er*	12.6±1.3	9.6±0.7	36.4±3.0	93.4±14.0	1.3	0.04	3.8	0.03	11
*ppd*	15.8±1.8	9.7±0.8	33.4±2.4	103.1±19.2	1.6	0.07	3.3	0.11	5
*jaw-D ppd*+/–	15.3±1.7	12.5±1.2	47.2±4.4	145.8±22.6	1.2	0.03	3.9	0.0	10
*35S::ICK2* (het)	7.1±1.1	5.8±0.9	21.7±3.3	33.2±10.7	1.2	0.04	3.8	0.04	6
*tni 35S::ICK2*	10.9±1.9	9.8±2.6	31.7±5.1	84.3±28.2	1.1	0.12	3.5	0.15	7
*ga1-3*	4.9±0.5	3.5±0.6	14.0±1.5	13.7±3.1	1.4	0.06	3.8	0.02	15
*tni ga1-3*	6.1±0.7	5.6±0.6	19.4±2.7	27.6±6.9	1.0	0.06	3.7	0.03	3
Col-0 (–PBZ)	11.4±1.0	8.3±0.6	34.5±2.2	80.4±4.8	1.3	0.06	3.8	0.06	6
Col-0 (+PBZ)	6.3±0.9	5.6±0.6	18.4±0.9	24.3±2.1	1.1	0.04	3.7	0.02	8
*tni* (–PBZ)	14.3±2.1	16.1±1.4	42.4±4.4	176.3±33.2	0.9	0.03	3.2	0.05	6
*tni* (+PBZ)	6.2±0.8	6.4±0.8	20.8±3.8	31.8±6.6	1.0	0.04	3.7	0.15	11

Mean values ± SEM are shown. Statistical significance of difference has been determined by Student’s *t*-test; *P*-values are given in Supplementary Table S2.

*For calculating the predicted perimeter and area from the measured length and width values at various growth stages, the following standard equations for an ellipse were used (with the assumption that the leaves are all planar ellipses):*

$$P=2\pi \surd \left(\frac{a^{2}+b^{2}}{2}\right)$$

and

A=πab

where *P* is the perimeter, *A* is area, *a* is half of the length and *b* is half of the width.

For the leaf growth rate experiment, the width of a given leaf of Col-0 and *tni* was first measured at emergence (<1mm long), and measurements were then made on alternate days for the next 20 days. The width of leaves >3mm long was measured using non-elastic stitching thread, and the absolute width was calculated using a ruler. The width of leaves <3mm long was measured using a thin copper wire with minimum graduation of 250 μm [graduation was made manually using a Rabone scale (UK)].

### Epidermal cell measurements

For measuring epidermal cell size, impressions of adaxial and abaxial surfaces of leaves were taken with dental wax (PRESIDENT, article no. 4667, Coltene, Switzerland). Casts were made with Araldite (Huntsman Advanced Materials Pvt. Ltd, India), using the dental wax on the moulds, and gold-coated with sputter coater (Jeol, Germany); these were loaded on to the stage of an ESEM Quanta 200 scanning electron microscope (Fei Company, USA). Images were taken at 25kV with a spot size of 3.0nm (the spot size is the space occupied by the cone of the electron beam on the sample surface) and working distance of 10mm. The average cell size per field was calculated by dividing field area by number of cells. A minimum eight fields were averaged to obtain the cell size in a leaf. Trichomes were photographed using a light microscope. For determining the frequency of trichome branching, mature, adaxial trichomes were observed with the light microscope, the number of branches was noted, and the frequency was expressed as a percentage of the total number of trichomes.

### Gus assay

Gus assay was performed as described previously (Sessions *et al.*, 1999). Samples were collected in 90% acetone on ice, incubated at room temperature for 30min and washed with staining buffer (50mM sodium phosphate pH 7.0, 0.2% Triton X-100, 10mM potassium ferrocyanide, 10mM potassium ferricyanide). Fresh staining buffer was added along with 1mM X-Gluc and vacuum infiltrated for 30min followed by incubation at 37°C for a maximum 90min. Staining buffer was replaced with 70% ethanol and this was incubated for 2h at room temperature; samples were then transferred to Hoyer’s medium (Anderson, 1954) and incubated for 1 day for clearing, following by mounting on glass slides. Observation and image acquisition were performed using an Olympus BX51 trinocular microscope (Olympus, Japan) fitted with ProgRes C3 camera using ProgRes Capture Pro 2.6 software. Multiple high resolution photographs were taken and merged in Adobe Photoshop CS3 to obtain panoramic view images of the entire leaf. For measuring the rate of progression of the mitotic arrest front in the distal-to-proximal direction, the entire leaf length and relative position of the arrest front were measured using Image J software.

For measuring the shape of the arrest front, equidistant, rectangular fields of 200×100 μm were demarcated within the mitotic arrest front from midrib to margin. Initially, in each field, the total numbers of epidermal and GUS-producing cells were manually counted, and the percentage of GUS-producing cells was calculated (Supplementary Figure S1A). For more objective measures, all the pictures were then run by iLASTIK, an open source image segmentation tool, to classify the pixels based on intensity (Sommer *et al.*, 2011). Segmentation of GUS-positive cells as one class and the rest as a background class was done based on selected features such as colour, edge, and texture (Supplementary Figure S1B). The images were then processed using CELL PROFILER software, with classification metadata input from iLASTIK, to identify GUS-positive cells (Jones *et al.*, 2009). In each field, total numbers of epidermal and GUS-producing cells were counted and the percentage of GUS-producing cells was calculated. The data analysed by the manual method and image segmentation tool produced similar results (Supplementary Figure S1C). When the measurements were carried out, the mitotic arrest front was at ~30–40% of the leaf length from the tip. It is important to note that the mitotic arrest front is not a sharp boundary. However, the shape of the arrest front did not change when measurements were taken at slightly different positions along the proximal-to-distal axis within the arrest front. Serrations were usually avoided while recording the measurements.

### Treatment of plants with paclobutrazol

For whole plant application, plants were treated with 120 µM paclobutrazol (PBZ; Duchefa Biochemie, The Netherlands) as described previously (Jacobsen and Olszewski, 1993). For treatment of a single leaf, 120 µM PBZ was applied on the fifth rosette leaf using a paint brush (Camlin, India; No. 00 with three-quarters of the bristles removed). Application started with the emergence of the leaf (~1mm long) and was continued 2–3 more times on alternate days. Mature leaves of untreated and treated plants were photographed and morphometric parameters were measured.

### DNA microarray experiments

Total RNA was isolated from 1–3mm long rosette leaves on the fifth node using the RNeasy Plant Mini Kit (Qiagen, Germany) from two different plant populations for biological replicates. The RNA samples were labelled with a single colour (Cy3) and hybridized on a 4×44 K *Arabidopsis* Agilent microarray chip (G2519F_021169); the intensity of spots was measured and normalized according to company specifications (Agilent Technologies, USA). The signal intensities of replicates were averaged and compared. The list of genes affected has been submitted to Gene Expression Omnibus (http://www.ncbi.nlm.nih.gov/geo/) with the accession number GSE38111. For a comparison of the *tni* gene expression profile with those under gibberellic acid (GA) treatment or with *tcp* mutants (Schommer *et al.*, 2008; Ribeiro *et al.*, 2012), publicly available expression data sets were obtained and analysed using Genevestigator tools (https://www.genevestigator.com).

## Results

### *tni* leaves produce larger, cup-shaped lamina

**Fig. 1.**
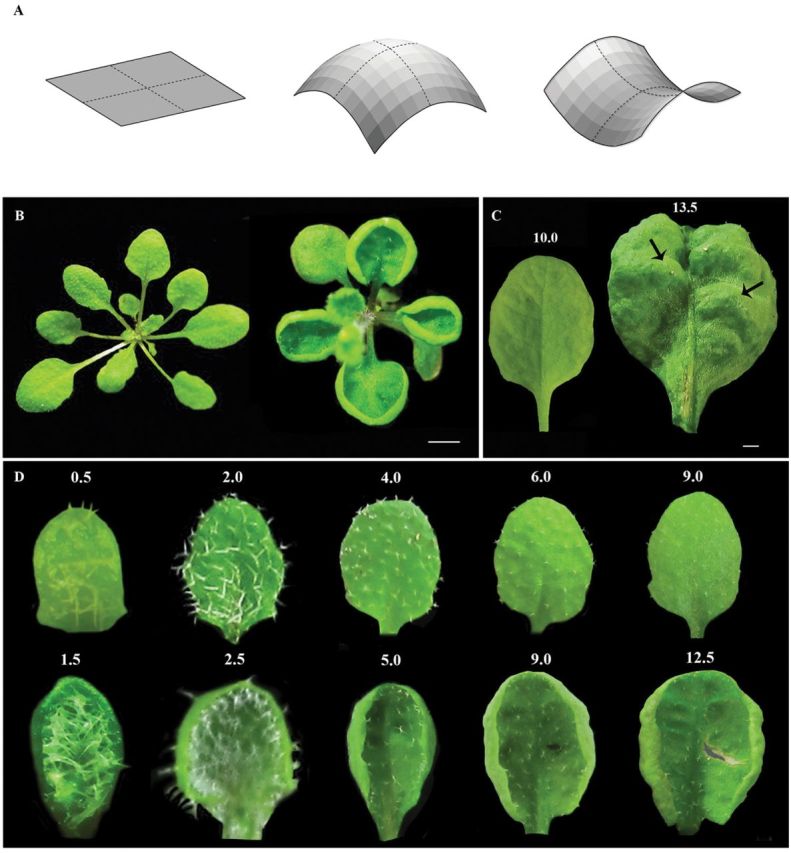
Phenotype of *tni* leaves. (A) Schematic representation of surfaces with zero (left), positive (middle), and negative (right) Gaussian curvature, which is measured by the product of linear curvatures along orthogonal axes (dotted lines). (B) 1-month-old Col-0 (left) and *tni* (right) rosettes showing differences in leaf shape. Scale bar = 1cm. (C) Abaxial view of mature Col-0 (left) and *tni* (right) leaves. Bulges in the *tni* lamina are indicated by arrows. Numbers indicate leaf length in mm. Scale bar = 5mm. (D) Adaxial views of Col-0 (top) and *tni* (bottom) leaves at various growth stages, highlighting differences in shape and curvature. Note that positive curvature in the *tni* lamina is already visible in leaves of 1.5mm length. Numbers above the leaves indicate leaf length in mm.

**Fig. 2.**
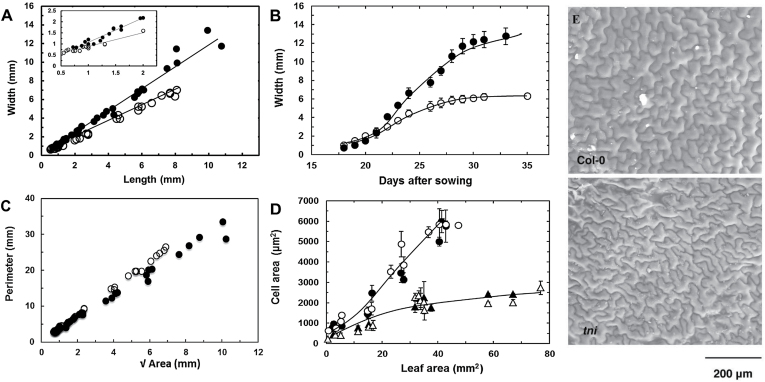
Morphometric analyses of the *tni* leaf. (A) Width and length of growing Col-0 (open circles) and *tni* (filled circles) leaves. The inset highlights shape differences at an early growth stage. (B) Growth kinetics of Col-0 (open circles) and *tni* (filled circles) leaves. (C) Leaf perimeter values are plotted against √Area for Col-0 (open circles) and *tni* (filled circles) leaves. When straight lines are fitted through the data points (not shown), the slopes (*P*/√*A*) are 3.72 for Col-0 and 3.07 for *tni*. (D) Average cell size of adaxial (open circles, open triangles) and abaxial (filled circles, filled triangles) surfaces of Col-0 (open circles, filled circles) and *tni* (open triangles, filled triangles) leaves. Error bars indicate SEM (*n* = 8–10 leaves). (E) Electron micrographs of adaxial epidermal cells of mature leaves.

The *tarani* (*tni*; ‘boat’ in Sanskrit) mutant was identified in a forward genetic screen aimed at isolating mutants with altered leaf shape. The recessive *tni* mutation mapped between 7.1 and 7.5Mb of chromosome 3. A mature *tni* leaf is larger, rounder, and curves upwards to form a cup-shaped lamina, while wild-type *Arabidopsis* leaves are flat with an ovoid shape (Fig. 1). To study the effect of the *tni* mutation on leaf shape, we measured the length and width of mature *tni* leaves and compared these with wild-type values. While Col-0 leaves on the fifth node from the base showed a length:width ratio of 1.4, *tni* leaves grew slightly more in length but almost twice as much in width, reducing the final length:width ratio to 0.9 (Table 1). This reduced leaf index value was established when the leaves were <1mm long (Fig. 2A), suggesting that the growth defect in the *tni* lamina is established early in development. Increased growth also resulted in a significantly larger *tni* leaf. While the average area of a mature Col-0 leaf was 90.3mm^2^, a mature *tni* leaf grew up to 180mm^2^ (Table 1). This excess growth resulted from a faster growth rate as well as longer length of growth in the medio-lateral direction, as measured by the change in width over time (Fig. 2B). At the fastest growth phase, the *tni* leaves grew at a rate twice that of the wild-type value of 0.5mm day^–1^. Growth kinetics of *tni* leaves along the length axis, however, were comparable to those of the wild type (Supplementary Figure S2).

Unlike Col-0 leaves, the cup-shaped *tni* leaves could not be flattened without introducing cuts in the margin (Supplementary Figure S3). A surface can in principle be flat or curved. The Gaussian curvature (Fig. 1A; Prusinkiewicz and Barbier de Reuille, 2010) provides a curvature measure, which is tightly related to lateral surface expansion. Young *tni* leaves form a cup-shaped lamina (Fig. 1D) with positive Gaussian surface curvature. At later stages of growth, the leaf margin folds back onto itself (Fig. 1B, D) making the surface curvature heterogeneous across the lamina. Since measuring the Gaussian curvature of such a surface is difficult, we used a proxy measurement of surface curvature by dividing the perimeter (*P*) with the square root of the area (√*A*). We are aware that this parameter (*P*/√*A*) suffers from inaccuracy since its value is dependent on the distribution of the Gaussian curvature across the surface, as well as on the shape in the planar dimension. Nevertheless, in the absence of a more accurate parameter, *P*/√*A* has been used prevously as a measure of leaf surface curvature (Nath *et al.*, 2003; White, 2006).

The values of the length:width ratio show that the *tni* leaves are less elliptical than the Col-0 leaves (Table 1). In order to determine to what extent this difference in planar shape would contribute to their *P*/√*A* values, we first calculated their theoretical *P*/√*A* values from the measured leaf length and width (see Materials and Methods). Both *tni* and Col-0 leaves showed similar values of ~3.6 (Supplementary Figure S4), suggesting that the difference in their planar shape has little effect on the *P*/√*A* parameter. We then determined the actual *P*/√*A* values from direct measurements of perimeter and area (Fig. 2C, Table 1; Supplementary Figure S4) and compared these between the two genotypes. Mature Col-0 leaves have flat laminae with an average perimeter of 36.3±1.6mm and a *P*/√*A* value of 3.8±0.3 (Table 1). The measured *P*/√*A* values of Col-0 were slightly higher than the predicted values at all growth stages (Supplementary Figure S4), perhaps due to an imperfect elliptical shape of wild-type leaves. Even though the average perimeter of mature *tni* leaves increased to 42.7±3.7mm, the *P*/√*A* value was reduced to 3.2±0.3, possibly due to excess medial growth compared to the margin. The *P*/√*A* of the Col-0 leaf was maintained at a constant value throughout the growth phase (Fig. 2C, Supplementary Figure S4)). However, although the *P*/√*A* value of the early *tni* leaf was comparable with the Col-0 value, it decreased progressively during later growth stages, suggesting that excess medial growth in the *tni* leaf continued throughout leaf morphogenesis.

### Cell proliferation is prolonged in *tni* leaves

To examine the basis of excess growth, we compared the epidermal cell size of *tni* leaves with that of Col-0 (Fig. 2D, E). The pavement cells of mature Col-0 leaves on the fifth node from the base grew to ~6000 µm^2^ while the *tni* pavement cells grew to ~3000 µm^2^. Thus, even though *tni* leaves are larger, they are made of more, smaller cells, suggesting excess cell proliferation. While an average mature Col-0 leaf contains ~15 000 pavement cells, a *tni* leaf had about four times more cells. As in Col-0, the *tni* pavement cells at the adaxial surface were of similar size to those at the abaxial surface at all growth phases (Fig. 2D). The frequency distribution of size of adaxial and abaxial epidermal cells, sampled throughout the surface of mature Col-0 and *tni* leaves, showed a similar trend (Supplementary Figure S5), i.e. in both Col-0 and *tni* leaves the smaller cells are more abundant in the abaxial surface whereas the larger cells are more abundant in the adaxial surface. Thus, it is plausible that the upward curvature of *tni* leaves does not arise from differential expansion of cells on the two surfaces. However, it should be kept in mind that a minor difference in cell size or cell proliferation rate between the adaxial and abaxial epidermal cells can cause upward bending of leaves. Nevertheless, when cell proliferation and mitotic expansion were reduced by overexpressing the cell division inhibitor *ICK2* (De Veylder *et al.*, 2001), the *tni* leaves became smaller with flat laminae (Table 1, Fig. 5), highlighting the role of cell proliferation in the *tni* phenotype.

**Fig. 3.**
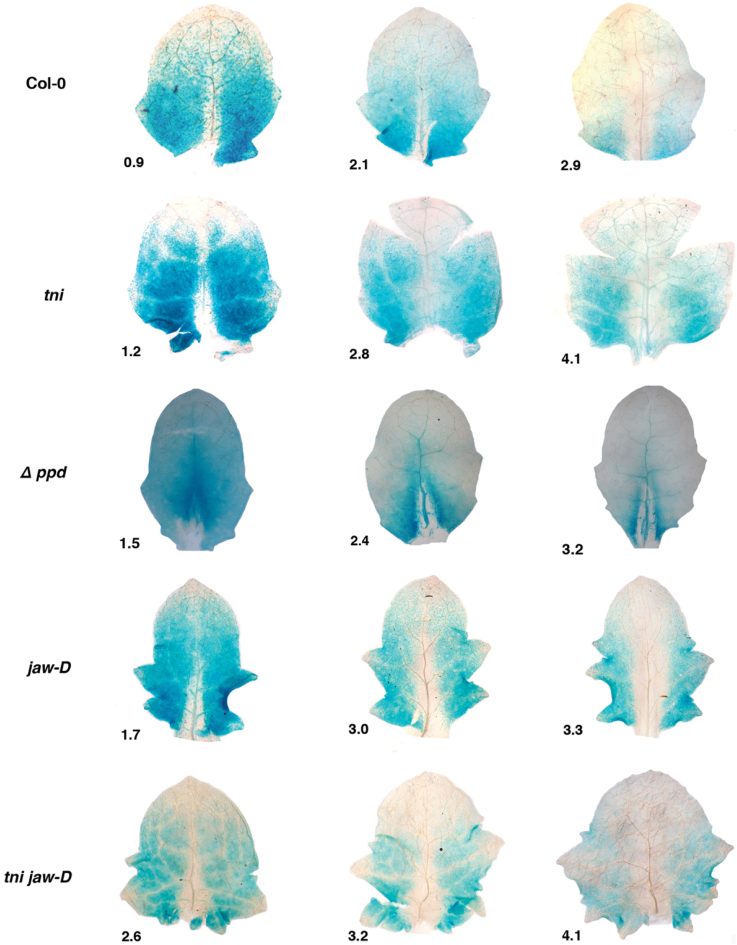
Cell proliferation activity in young leaves. *CYCLIN D3;2* expression is shown as GUS reporter activity (blue dots) in young leaves of the genotypes indicated. Numbers denote leaf length in mm.

Given that the proliferation zone remains anchored at the leaf base throughout the growth phase (Andriankaja *et al.*, 2012), its size and the dynamics of cell exit from this zone are likely to influence leaf shape and size (Powell and Lenhard, 2012). *CIN*-like *TCP* genes accelerate leaf maturation, possibly by restricting the mitotic zone and/or promoting cell exit from this zone (Nath *et al.*, 2003; Efroni *et al.*, 2008). To examine the dynamics of cell proliferation in *tni* leaves, we measured the cell division pattern in young Col-0 and *tni* leaves expressing the GUS reporter under the transcriptional regulation of *CYCLIN D3;2*, a gene encoding a mitotic cyclin required for developmentally regulated cell division (Dewitte *et al.*, 2007). In a 0.9mm long Col-0 leaf, transcription of *CYCLIN D3;2* was observed throughout the lamina except at the distal region and midrib (Fig. 3). Expression was progressively restricted towards the base as the leaf grew, and little expression was observed at the base of a ~3mm long leaf. A mitotic arrest zone was also observed at the tip of a young *tni* leaf, which expanded more slowly than in Col-0 (Fig. 3). Reduced mitotic activity was also observed at the mid-vein and near the vasculature of *tni* leaves.

**Fig. 4.**
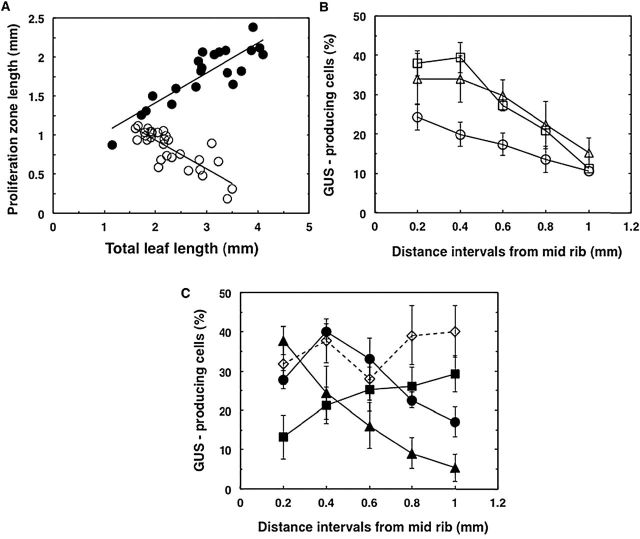
Cell proliferation dynamics along the orthogonal axes of young leaves. (A) Length of proliferation zone plotted against total length of Col-0 (open circles) and *tni* (filled circles) leaves. Each data point represents a measurement from a single leaf. A straight line was fitted to the data points (*R* ^2^ is 0.67 for Col-0 and 0.76 for *tni*) and the slope values for Col-0 and *tni* were –0.37 and +0.43mm^–1^, respectively. (B, C) Percentage of GUS-producing cells in the transition zone (refer to Supplementary Figure S1) is plotted against distance from midrib to margin of Col-0 (open circles), *arf2-*8 (open squares), and *klu-4* (open triangles) (in B) and of *tni* (filled circles), *jaw-D* (filled squares), *Δppd* (filled triangles), and *tni jaw-D* (open diamonds) (in C). 100–150 pavement cells were counted per data point per region of leaf. Mean values of 11 leaves in Col-0 and five leaves for other genotypes are shown. Leaf length varied from 1.7 to 2.2mm (Col-0 and *klu-4*), 2.3 to 2.8mm (*tni*), 2.8 to 3.3mm (*jaw-D*), 1.9 to 2.6mm (Δ*ppd* and *arf2-8*), and 3.2 to 4.0mm (*tni jaw-D*). The mitotic arrest front was at ~one-third of the leaf length from the tip when the analysis was carried out. Error bars are SEM.

To compare the dynamics of cell proliferation in *tni* and Col-0 leaves, we measured the length of the proliferation zone proximal to the arrest front. In a developing leaf, the transition from the distal arrested zone and the proximal proliferating zone is gradual and no boundary can be drawn between the two (Fig. 3) (Nath *et al.*, 2003; Efroni *et al.*, 2008; Andriankaja *et al*., 2012). Therefore, the proliferation zone was arbitrarily defined as the distance between the base of the lamina and the transition zone where ~10% of adaxial cells showed GUS activity adjacent to the midrib. In Col-0, for every 1mm growth in total leaf length, the proliferation zone decreased by 0.37mm (Fig. 4A). In contrast, the *tni* proliferation zone first increased by 0.38mm for every 1mm growth in total leaf length and then sharply decreased (data not shown).

### Cell proliferation pattern along the medio-lateral axis is altered in *tni* leaves

In young *cin* leaves, the shape of the mitotic arrest front within the transition zone is strongly concave, leading to more growth in the margin and crinkly laminae. One may then predict that changing the shape of the arrest front to highly convex would lead to excess growth in the centre compared to the margin, resulting in a cup-shaped leaf (Sarvepalli and Nath, 2011). To examine whether the shape of the arrest front is changed in the *tni* leaf compared to Col-0, we compared cell proliferation between the medial region and margin. We estimated the mitotic density by measuring the percentage of GUS-producing cells, which reports *CYCLIN D3;2* expression, along the medio-lateral axis within the transition zone and expressed it as a function of distance from the midrib. In young Col-0 leaves, ~25% of cells near the midrib expressed GUS and the value progressively decreased to 10% near the margin (Fig. 4B, Supplementary Figure S1C), giving a mild convex shape to the mitotic arrest front, as was observed in young *Antirrhinum* leaves (Nath *et al.*, 2003). The mitotic arrest front showed similar overall shape when measured at various places within the broad transition zone (data not shown). A progressive decrease in mitotic cell density from the medial region to the margin was also observed in the flat leaves of mutant lines where leaf size was either increased (*auxin response factor2-8*) or decreased (*klu-4*) (Fig. 4B; Supplementary Figures S6, S7; Schruff *et al.*, 2006; Anastasiou *et al.*, 2007), suggesting that perturbed cell division per se does not lead to a change in the shape of the mitotic arrest front.

In young *tni* leaves, ~27% of cells produced the GUS reporter near the midrib within the mitotic arrest front. However, the proportion of the GUS-producing cells increased to ~40% slightly towards the margin and then decreased to ~15% at the extreme margin (Fig. 4C), giving the arrest front a strong convex shape with a dip at the midrib. The *Δppd* leaves which produce downward-curving, cup-shaped lamina (White, 2006) also showed a strong convex-shaped arrest front (Figs. 3, 4C). A strongly convex-shaped mitotic arrest front is expected to result in excess mature cells around the medial region compared with the margin, leading to cup-shaped leaves. In contrast to *tni* and *Δppd* leaves, the arrest front in the *jaw-D* leaf was concave, which might lead to more cell proliferation and mitotic expansion in the margin than the centre, consistent with its negative surface curvature (Figs. 3, 4C). These results possibly imply a link between varying cell division patterns along the medio-lateral axis and curvature differences in diverse genetic backgrounds.

### *TNI* maintains leaf flatness independent of *CIN*-like *TCP* genes and *PEAPOD*s

**Fig. 5.**
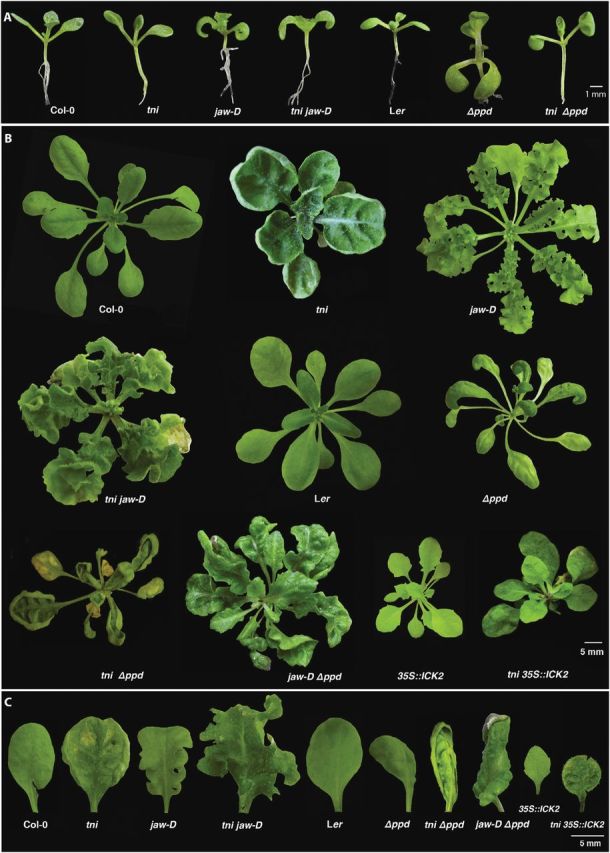
Genetic interactions of *tni* with other curvature mutants. (A) 12-day-old seedlings of the genotypes indicated highlighting the cotyledon epinasty phenotype and its rescue. (B) 25–40-day-old rosettes of the genotypes indicated. The *35S::ICK2* plant was heterozygous for the transgene. The *tni 35S::ICK2* individual had at least one copy of the *35S::ICK2* transgene. (C) Mature leaves on the fifth node of the genotypes indicated are shown.

**Fig. 6.**
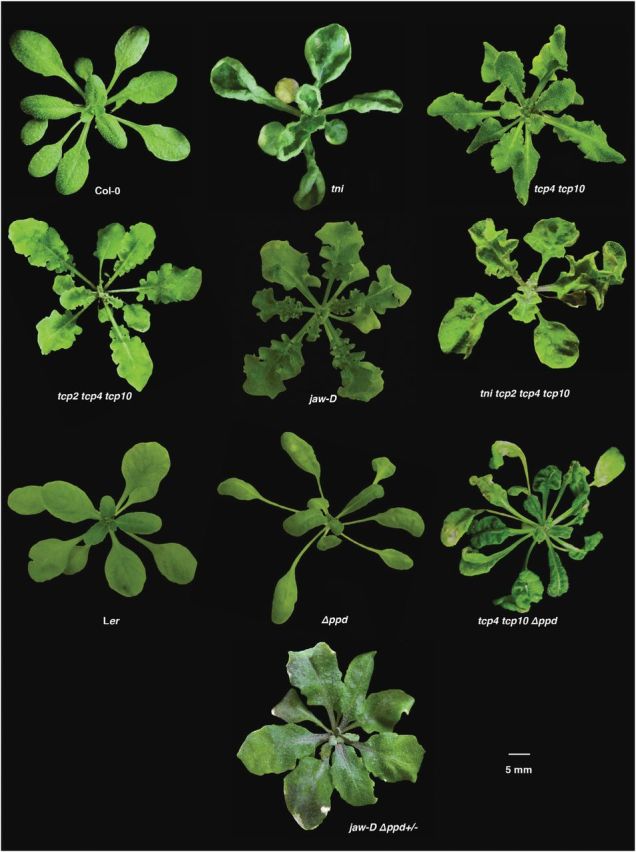
Genetic interactions among surface curvature mutants. 30–35-day-old rosettes of the genotypes indicated are shown. Note that the highly crinkly phenotype of *jaw-D* leaves is rescued to almost flat in the *Δppd/+* background.

When five *CIN*-like *TCP* genes were downregulated by overexpressing the micro RNA MIR319 in the *jaw-D* line, or by combining all the loss-of-function mutations, the resultant plants showed a crinkled leaf margin (Palatnik *et al.*, 2003; Schommer *et al.*, 2008; Koyama *et al.*, 2010). Differentiation was delayed in these leaves and the progression of the mitotic arrest front was slowed (Efroni *et al.*, 2008). To determine whether *TNI* controls leaf flatness independent of *TCP* and *PPD* genes, we generated mutant combinations by crossing *tni* with *jaw-D*, *tcp2 tcp4 tcp10*, and *Δppd* and studied their shape parameters (Figs. 5, 6; Table 1). The *jaw-D* leaves showed a slight increase in both length and width compared to the wild type, without altering the leaf index value. The length of *jaw-D* was also comparable to that of the *tni jaw-D* double mutant, suggesting a minor role played by these two genes affecting growth in the proximo-distal axis. In contrast, the width of the *jaw-D* leaf was increased by >60% in the *tni* mutant background, suggesting that *TNI* controls growth in the medio-lateral axis independent of the *TCP* genes. Consequently, the leaf index value of the *tni jaw-D* line was reduced to 1.01, much less than the *jaw-D* value.

Although the perimeter and area of *jaw-D* leaves increased by ~30% compared to Col-0, *P*/√*A* increased to 4.3 compared with the Col-0 value of 3.8 (Table 1), possibly indicating excess margin growth (Nath *et al.*, 2003). In the *tni jaw-D* leaves, width, perimeter, and area exceeded the values of the single mutants. The *tni jaw-D* leaves were more crinkly than the *jaw-D* single mutant, with a net surface curvature value of 5.3, possibly due to excess growth (Nath *et al.*, 2003). The shape of the mitotic arrest front of young *tni jaw-D* leaves appears to show a combination of those in respective single mutants (Figs. 3, 4C), possibly implying that *TNI* and *TCP* genes act independently. The *tni tcp2 tcp4 tcp10* quadruple mutant also showed phenotypic features similar to the *tni jaw-D* mutant (Fig. 6), demonstrating that the *tni jaw-D* phenotype was indeed due to combined loss of *TNI* and *TCP* function and not due to a *TCP*-independent overexpression effect of miR319 (Palatnik *et al.*, 2003). The *tni Δppd* double mutant also showed features of both *tni* and *Δppd* laminae, with a combination of up- and downward folds (Fig. 5), suggesting an independent function of these two genes.

When the *Δppd* mutant was crossed with *jaw-D*, the resultant *jaw-D Δppd* double homozygous plants produced leaves with phenotypes that were intermediate between the single mutants (Fig. 5). Interestingly, *Δppd*/*+* completely rescued the crinkliness of the *jaw-D* leaves and produced flat *Δppd*/*+ jaw-D* leaves that resembled Col-0 (Fig. 6; Table 1), reiterating the dominant nature of the *Δppd* mutation (White, 2006). When *Δppd* was crossed with the *tcp4 tcp10* double mutant, which produces mildly crinkly leaves, the resultant triple mutant (*Δppd tcp4 tcp10*) also produced leaves with features of both parental lines (Fig. 6). Taken together, these results show that the *TNI*, *TCP*, and *PPD* genes maintain leaf flatness independent of one another.

### Reduction of endogenous GA levels rescue the *tni* phenotype

**Fig. 7.**
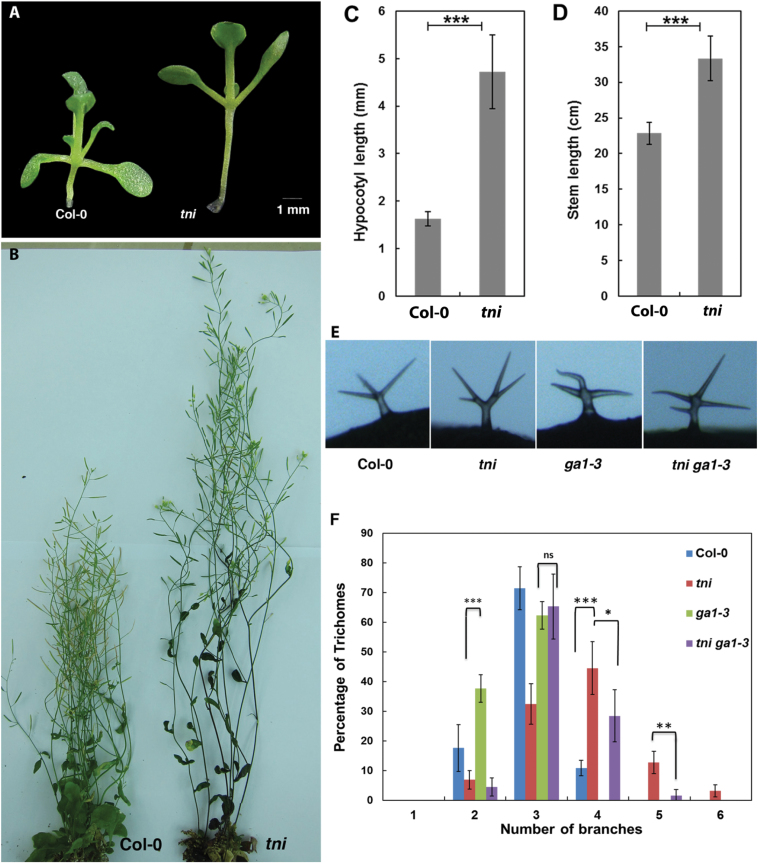
GA-related phenotypes of the *tni* mutant. (A, B) Seedlings from the root–shoot junction and above (A) and mature plants (B) of Col-0 and *tni* are shown, highlighting the elongated hypocotyl and stem in *tni*. (C, D) Plots of hypocotyl length (C) averaged over 29 seedlings each in Col-0 and *tni*, and stem length (D) averaged over 13 seedlings each in Col-0 and *tni*. Error bars represent SEM. ***, *P* ≤ 0.001. Student’s *t*-test was used. (E) Microscope photographs of representative trichomes of the genotypes indicated highlighting altered branching phenotypes. (F) Frequency of trichomes (as a percentage of the total) of genotypes indicated plotted as a function of trichome branch numbers. 297–712 trichomes from 3–6 mature leaves were scored for their branch numbers and plotted. Error bars represent SEM. ***, *P* ≤ 0.001; **, *P* ≤ 0.01; *, *P* ≤ 0.05. Student’s *t*-test was used.

**Fig. 8.**
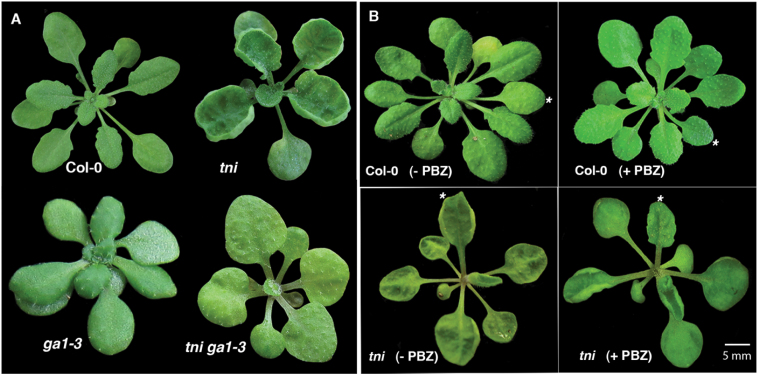
Reduction in GA level rescues the *tni* leaf phenotype. (A) Rosettes of the genotypes indicated are shown. Note the rescue of *tni* leaf curvature in the *ga1-3* background. (B) 1-month-old rosettes of Col-0 and *tni* plants with (+PBZ) or without (–PBZ) PBZ treatment on the fifth leaf (*). Note the rescue of positive curvature only in the fifth *tni* leaf.

Distribution of endogenous GA in a young leaf is closely associated with leaf morphogenesis in diverse plant species (Achard *et al.*, 2009; Nelissen *et al.*, 2012). GA regulates several aspects of *Arabidopsis* development, including cell proliferation, expansion, endoreduplication-mediated trichome branching, and flowering (Wilson *et al.*, 1992; Chien and Sussex, 1996; Jacobsen *et al.*, 1996), consistent with its ubiquitous distribution and activity (Silverstone *et al.*, 1997; Tyler *et al.*, 2004). The *tni* plants display features resembling elevated GA levels/signalling, such as increased length of the hypocotyl and stem, fewer rosette leaves, and hyper-branched trichomes (Fig. 7; Supplementary Figure S8; Silverstone *et al.*, 2007). To determine whether *tni* leaf shape is associated with altered GA levels, we reduced its endogenous GA level by crossing it with the GA-deficient mutant *ga1-3* (Sun *et al.*, 1992). The *ga1-3* plants produce smaller leaves with flat laminae. The *tni ga1-3* double mutant also produced flat leaves (Fig. 8A), although leaf area decreased compared to *tni* (Table 1). The rescue of *tni* leaf curvature by the *ga1-3* mutation was not due to a change in leaf size, since curvature rescue was not observed when leaf size was either reduced or increased independently in the *tni klu-4* or *tni arf2-8* double mutants, respectively (Supplementary Figure S9). The *ga1-3* mutant also rescued other *tni* phenotypes, including trichome hyper-branching (Fig. 7E, F). Rescue of *tni* curvature was independently achieved by external application of the GA antagonist paclobutrazol (PBZ). Both systemic (Supplementary Figure S10) and local (Fig. 8B) applications of PBZ on young *tni* leaves resulted in rescue of positive curvature, with the *P*/√*A* value restored to its Col-0 level (Table 1). Taken together, reduction of endogenous GA levels partly rescued the *tni* leaf phenotype.

### Global transcriptome analysis of young *tni* leaves

In the absence of *TNI* identity, we attempted to gain insight into *TNI* function by comparing the global transcripts of young *tni* leaves with those of Col-0 by DNA microarray analysis. A total of 770 genes were upregulated and 944 genes downregulated by ≥2-fold in *tni* leaves (Supplementary Figure S11). The genes that were significantly upregulated in *tni* leaves were involved with the cell cycle, proteolysis, RNA processing, and transposons. Eighteen cell cycle-related genes were upregulated in the *tni* mutant, which did not include cyclins or cyclin-dependent kinases. Genes preferentially downregulated in the *tni* mutant were involved with signalling processes, cell wall modification, general metabolism, stress, and phytohormones.

Since the *tni* phenotype resembled an elevated GA phenotype in several aspects (Fig. 7), we compared the transcripts differentially regulated in *tni* leaves with those in GA-treated seedlings that are available in the public domain (Ribeiro *et al.*, 2012). We did not observe any significant similarity between the overall transcript changes between *tni* and GA-treated plants (Supplementary Table S3). While expression of 125 genes changed in a similar way in *tni* and GA-treated plants, expression of 214 genes showed opposite expression changes in *tni* and GA-treated leaves. We also compared the *tni* transcriptome profile with that in *jaw-D* and *TCP* gain-of-function lines, since cell proliferation is deregulated in both *tni* and *tcp* leaves (Efroni *et al.*, 2008). Similarly, *tni* microarray data was compared to that of *TCP* mutants (*tcp2;tcp4*) and *rTCP4* (Schommer *et al.*, 2008); little similarity was found between the transcriptome profiles.

## Discussion

Leaf growth is a result of cellular expansion during cell proliferation and differentiation. Genes that promote proliferation are active throughout the lamina at a very early growth stage (Horiguchi *et al.*, 2005; Rodriguez *et al.*, 2010) and produce adequate numbers of cells. Recent leaf growth models show that the zone of proliferation remains anchored at the base and its size stays more or less constant throughout the growth phase (Andriankaja *et al.*, 2012). The newly divided cells are pushed away from the proliferation zone towards the leaf tip to form an arrest zone, where they lose their proliferation potential and enter into a phase of differentiation-associated expansion. As more and more cells enter the differentiation phase, the arrest zone increases in size, reducing the relative proportion of the proliferation zone. The proliferation zone then disappears rapidly, terminating cell division and eventually leaf growth (Andriankaja *et al.*, 2012). Several factors, such as the size of the proliferation zone and the duration of its activity, the rate of cell exit into the arrest zone, and the rate of cell division and expansion, are likely to have profound effect on the relative area occupied by these two regions, and consequently on the final leaf size (Gonzalez *et al.*, 2012; Powell and Lenhard, 2012). A premature termination of the proliferation zone would result in a smaller leaf with fewer cells, whereas its prolonged presence would lead to a bigger leaf with excess cells (Mizukami and Fischer, 2000; Nath *et al.*, 2003; White, 2006; Efroni *et al.*, 2008; Sarvepalli and Nath, 2011). The *TNI* gene possibly restricts the size as well as the duration of the proliferation zone (Figs. 3, 4A) and thereby acts as a negative regulator of leaf growth. A smaller arrest zone during early growth of *tni* leaves may arise from reduced cell expansion (Fig. 2D, E) and fewer cells exiting the proliferation zone.

Kinematic analysis of root (Beemster and Baskin, 1998; Beemster *et al.*, 2002; Silk, 2006) and leaf growth (De Veylder *et al.*, 2001; Beemster *et al.*, 2005; Asl *et al.*, 2011) in wild-type *Arabidopsis* has uncovered the relationship between organ growth and the division/expansion of cells. Our analysis, however, does not clarify the exact role of *TNI* on cell division and expansion. A larger proliferation zone in *tni* leaves may result from increased proliferation-promoting mobile signal at the leaf blade/petiole junction (Kazama *et al.*, 2010), elevated cell division rate, or a reduction in the rate of cell exit into the arrest zone. In *Arabidopsis*, the duration of the cell cycle remains relatively constant throughout leaf development (Asl *et al.*, 2011). In most mutant leaves with increased cell proliferation, such as *cin*, *jaw-D*, and *35S::ANT*, the duration of proliferation is increased with no significant change in cell cycle rate (Mizukami and Fischer, 2000; Nath *et al.*, 2003; Efroni *et al.*, 2008). By contrast, leaf growth rate as well as duration increased in *tni* leaves (Fig. 2B). Whether *TNI* affects the rates of cell division and cell expansion can be resolved only by direct measurements using time-lapse microscopy.

Surface curvature in the leaf is expected to be influenced by regulated growth between the margin and medial regions. There are no significant margin-to-medial differences in cell division between the distal- arrested zone and the proximal division zone. However, the interfacing transition region might play an important role in maintaining leaf flatness. Within the transition region of the wild-type leaf, cell division in the medial region is slightly higher than at the margin, giving the so-called mitotic arrest front a slightly convex shape (Fig. 4B) (Nath *et al.*, 2003). In the *tni* leaf, differences in cell division and associated cell expansion between the medial and margin of the transition zone become stronger. If cell expansion is not differentially affected between these two regions due to compensation, this may result in excess medial growth and possibly in positive Gaussian curvature. *TNI*-mediated control of differential cell proliferation is independent of *TCP* genes (Figs. 4C, 5), mutation in which causes excess cell division at the margin. *TCP*-mediated suppression of marginal division is perhaps stronger than *TNI*-mediated suppression of cell division at the medial region, leading to a resultant negative Gaussian curvature in the *tni jaw-D* double mutant leaf (Fig. 5B, Table 1).

It is interesting to note that even though both *tni* and *Δppd* leaves make cup-shaped laminae, the former produce upwardly curving leaves while the latter make downwardly curving leaves. While the reason for this difference is not apparent from the data presented here, it is possible that small differences in cell proliferation or expansion rates between the adaxial and abaxial surfaces bring about the opposite effects. Detailed kinematic studies of cell division/expansion in these mutant leaves may uncover this difference. Another reason may be that the *PPD* genes control the proliferation of dispersed meristemoid cells (White, 2006), the density of which may vary between the adaxial and abxial surfaces. The role of *TNI* on meristemoid cell proliferation has not been observed.

From the analysis of mutants in genes involved in leaf morphogenesis, it is becoming increasingly clear that the control of cessation of cell proliferation, and the transition from mitotic to differentiation-associated expansion, is of prime importance in maintaining leaf shape and size. Marked effects on size and shape are observed when genes that restrict cell proliferation, such as *TCP*, *TNI*, and *PPD*, are mutated. Downregulation of *TCP* and *TNI* genes produces leaves with larger area, while their overactivation drastically reduces leaf size and cell number (Koyama *et al.*, 2010; Sarvepalli and Nath, 2011). More dramatic effects could be observed when two genes were mutated together in combinations of double mutants (Figs. 5, 6; Table 1), suggesting that these genes suppress leaf cell proliferation independently of one another. Their domains of action appear to be complementary as well; while *TCP* genes suppress cell proliferation and mitotic expansion primarily at the margin, *TNI* does so more at the centre (Figs. 5, 6), and consequently their mutations cause opposite leaf curvatures. Leaf flatness possibly depends on a delicate balance of the activities of these genes. Loss of both *TNI* and *TCP* genes does not result in a flat leaf, possibly due to a stronger deregulation of cell proliferation and mitotic expansion at the margin by the *jaw-D* mutation than that at the centre by the *tni* mutation.

Heterochronic factors such as *CIN*-like *TCP* genes advance the timing of the transition from proliferation to expansion (Nath *et al.*, 2003; Efroni *et al.*, 2008; Sarvepalli and Nath, 2011). Growth continues for a longer duration in the *tni* leaves as well (Fig. 2), presumably due to a more persistent and wider cell proliferation zone at the leaf base (Fig. 3, 4C). In this regard, *TNI* acts as a heterochronic gene in leaf maturation. However, faster growth of the *tni* leaf suggests a more direct role of *TNI* in cell proliferation and mitotic expansion (Figs. 2B, 3), which could result from elevated cyclin function. Increased growth rate has been reported in plants overexpressing *CYCLIN D2* (Cockcroft *et al.*, 2000). Interestingly, excess cell division in the *tni* mutant results in differential growth in two orthogonal axes: more in the medio-lateral axis than in proximo-distal axis (Table 1). It will be interesting to examine whether this polar preference in cell division and anisotropy of mitotic expansion is related to a change in the orientation of the plane of cell division.

The identity of the *TNI* gene is not yet known. The effect of the *tni* mutation on cell proliferation suggests that *TNI* encodes a cell division inhibitor that reduces the rate of the cell cycle in a spatially regulated manner. Faster growth rate and prolonged *CYCLIN D3;2* expression is observed in young *tni* leaves. Further, a general reduction of cell proliferation by overexpressing the cell division inhibitor *ICK2* partly rescued the *tni* phenotype. Super-numerary branches in *tni* trichomes (Figs. 7E, F) also indicate elevated CYCLIN function in the mutant, since overexpression of B and D cyclins can increase trichome branching via elevated endoreduplication (Ishida *et al.*, 2008). However, we did not observe an increased level of any *CYCLIN* or *CDK* transcripts in the *tni* leaves by global transcript analysis. Therefore, it is possible that *TNI* restricts cell division not by transcriptional control of the cell cycle factors, but through post-translational modification or degradation of the target proteins. The link between *TNI* function and the cell cycle could also be more indirect and through plant hormones (Koyama *et al.*, 2010; Yanai *et al.*, 2011). GAs have been implicated in promoting cell proliferation in growing *Arabidopsis* leaves (Achard *et al.*, 2009), and reduction in the endogenous GA level can rescue the *tni* leaf phenotype (Fig. 8, Table 1). Mapping the *tni* locus shows that *TNI* is unlikely to encode a protein involved in GA biosynthesis or signalling (data not shown). It is more likely that the *TNI* product acts upstream of GA. Interestingly, a transcript encoding a GA2ox4 (*AT1G02400*), an enzyme that inactivates GA, is downregulated 11-fold in *tni* leaves in our microarray experiment, suggesting an increased GA level. However, we do not observe significant similarity between the *tni* transcriptome profile and the leaf transcriptome change upon GA treatment (Supplementary Table S3). It is possible that the exogenous application of GA brings about transcriptome changes different from its endogenous counterpart. The mechanism of *TNI* function and its link to the GA pathway will perhaps be clearer when the *TNI* gene is cloned and its identity is determined.

## Supplementary material

Supplementary data can be found at *JXB* online.

Supplementary Table S1. Sequences of primers used in this study.

Supplementary Table S2. The *P*-values of the Student’s *t*-test carried out on the data shown in Table 1.

Supplementary Table S3. Comparison of transcriptome profiles.

Supplementary Figure S1. GUS expression along the medio-lateral axis.

Supplementary Figure S2. Growth kinetics of Col-0 and *tni* leaf length.

Supplementary Figure S3. Flattening of *tni* leaves by cutting.

Supplementary Figure S4. Comparison of predicted and measured *P*/√*A* values of Col-0 and *tni* leaves.

Supplementary Figure S5. Frequency distributions of epidermal cell size.

Supplementary Figure S6. Mutants with altered leaf size in *Arabidopsis*.

Supplementary Figure S7. The mitotic arrest zone in mutants with altered leaf size.

Supplementary Figure S8. Flowering time of Col-0 and *tni*.

Supplementary Figure S9. Surface curvature of the *tni* leaf is independent of leaf size.

Supplementary Figure S10. Rescue of the *tni* leaf phenotype by systemic application of PBZ.

Supplementary Figure S11. Global transcriptome analysis of *tni* leaves.

## Funding

PK and KRC were supported by fellowships from the Indian Institute of Science, Bangalore; UN was supported by a grant from the Department of Biotechnology, Govt of India (*BT/PR13149/BRB/10/739/2009*).

## Supplementary Material

Supplementary Data
