# Supplementary material for: The tarani mutation alters surface curvature in Arabidopsis leaves by perturbing the patterns of surface expansion and cell division
Source: J Exp Bot. 2015 Feb 24;66(7):2107–22. doi: 10.1093/jxb/erv015 (PMC4378639; doi:10.1093/jxb/erv015)
Supplement: Supplementary Data [file supp_66_7_2107__index.html]

The tarani mutation alters surface curvature in Arabidopsis leaves by perturbing the patterns of surface expansion and cell division — Supplementary Data 

# The *tarani* mutation alters surface curvature in *Arabidopsis* leaves by perturbing the patterns of surface expansion and cell division

## Supplementary Data

Data files

**Files in this Data Supplement:**

- Supplementary Data - Supplementary Data
