# Supplementary material for: The tarani mutation alters surface curvature in Arabidopsis leaves by perturbing the patterns of surface expansion and cell division
Source: J Exp Bot. 2015 Feb 24;66(7):2107–22. doi: 10.1093/jxb/erv015 (PMC4378639; doi:10.1093/jxb/erv015)
Supplement: Supplementary Data [file supp_erv015_erv015_Supplementary__Information_Corrected.pdf]

The *tarani* mutation alters surface curvature in *Arabidopsis* leaves by perturbing the patterns of surface expansion and cell division

Premananda, K., Krishna Reddy Challa and Utpal Nath

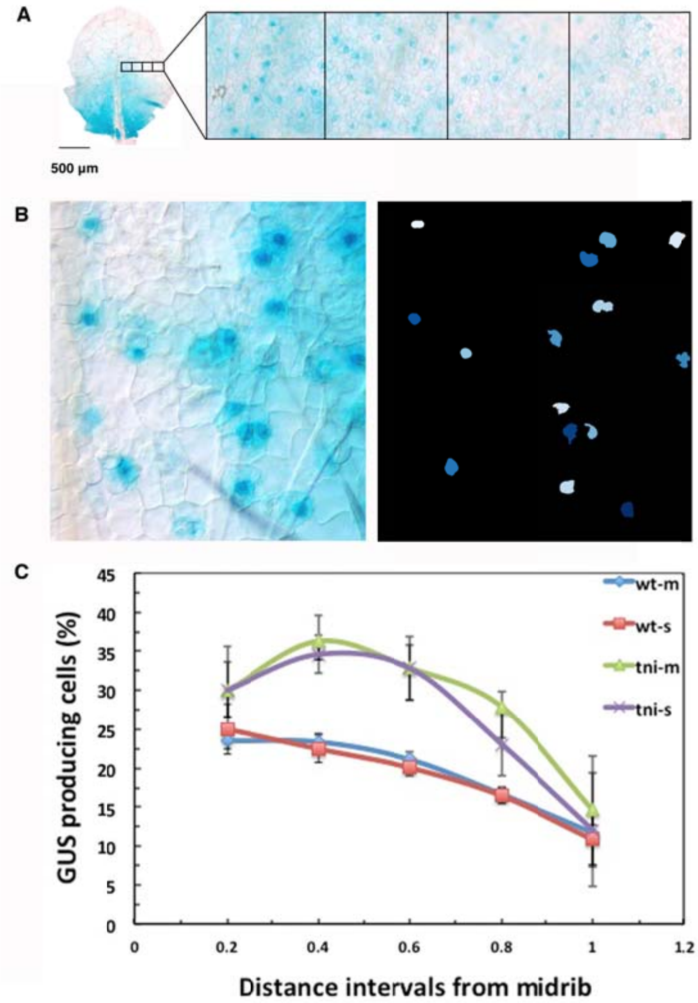

Fig. S1. GUS-expression along the medio-lateral axis. **A**. Left, a young Col-0 leaf expressing GUS reporter (blue colour). Right, the mitotic arrest front is magnified from mid-rib to margin to highlight the GUS-producing cells. Note a gradient of GUS-activity from medial to margin of the leaf. **B**. Left, a bright field, high resolution picture segment shown in **A**. Right, the same picture segment is shown after running it through ilastik and CellProfiler software and extracting the GUS-producing cells (light to dark blue dots). **C**. Percentage of GUS-producing cells in the transition zone is plotted against distance from midrib to margin of Col-0 (wt-m, wt-s) and of *tni* (tni-m, tni-s) as analyzed by manual observation of the blue dots (wt-m, tni-m) and by the software processing described above (wt-s, tni-s). Experimental conditions are similar to what is described in Figs. 4C, D. Error bars are SEM.

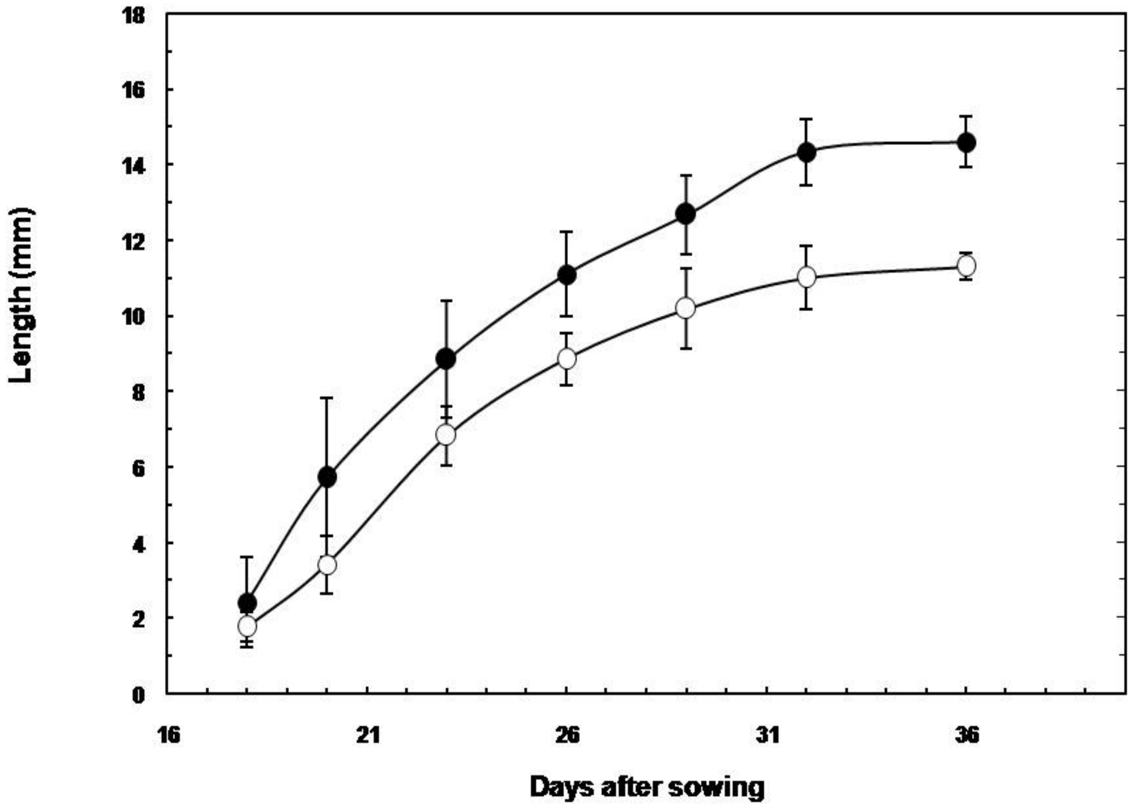

Fig. S2. Growth kinetics of Col-0 (○) and *tni* (●) leaf length. At the exponential growth phase, the growth rates for Col-0 and *tni* are 1.02 and 1.26 mm/day, respectively. The growth rates are not significantly different (p=0.09), as tested by Student's t-test.Sample numbers are 14 (Col-0) and 7 (*tni*).

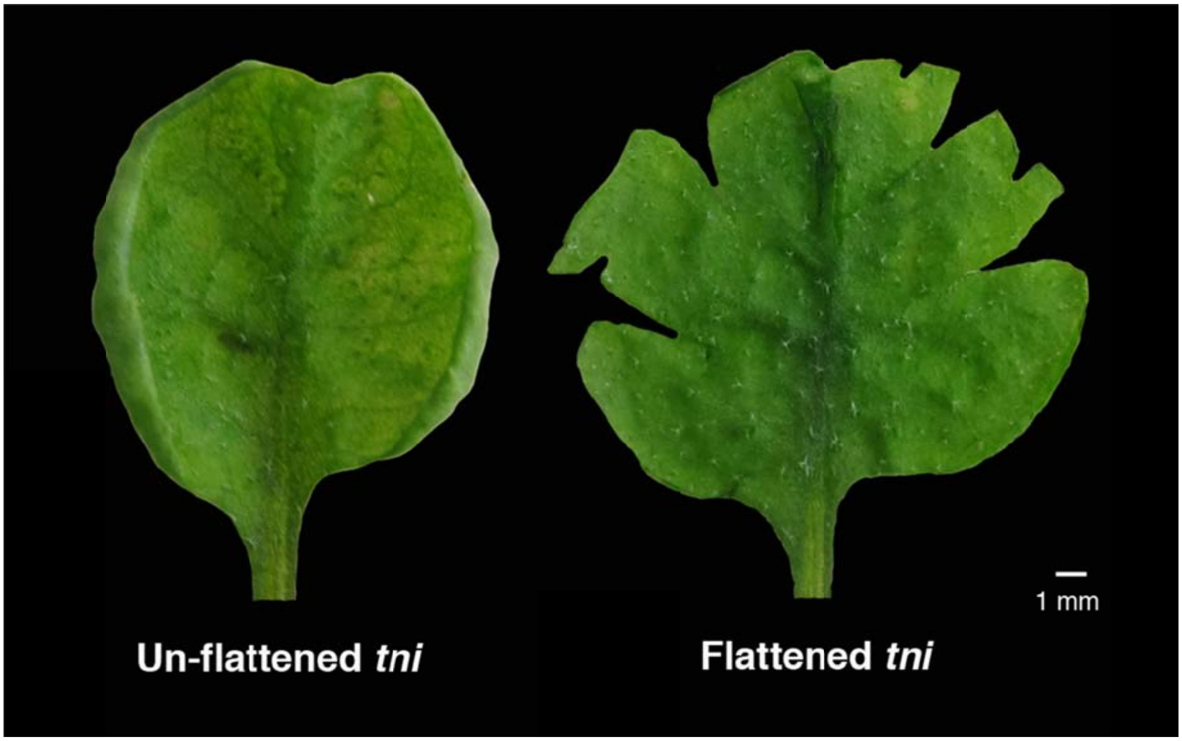

Fig. S3. *tni* leaves cannot be flattened without cuts.A mature *tni* leaf on the 5th node from the base is shown before and after flattening. Cuts were made at the margin before flattening, which resulted in gaps in the flattened leaf.

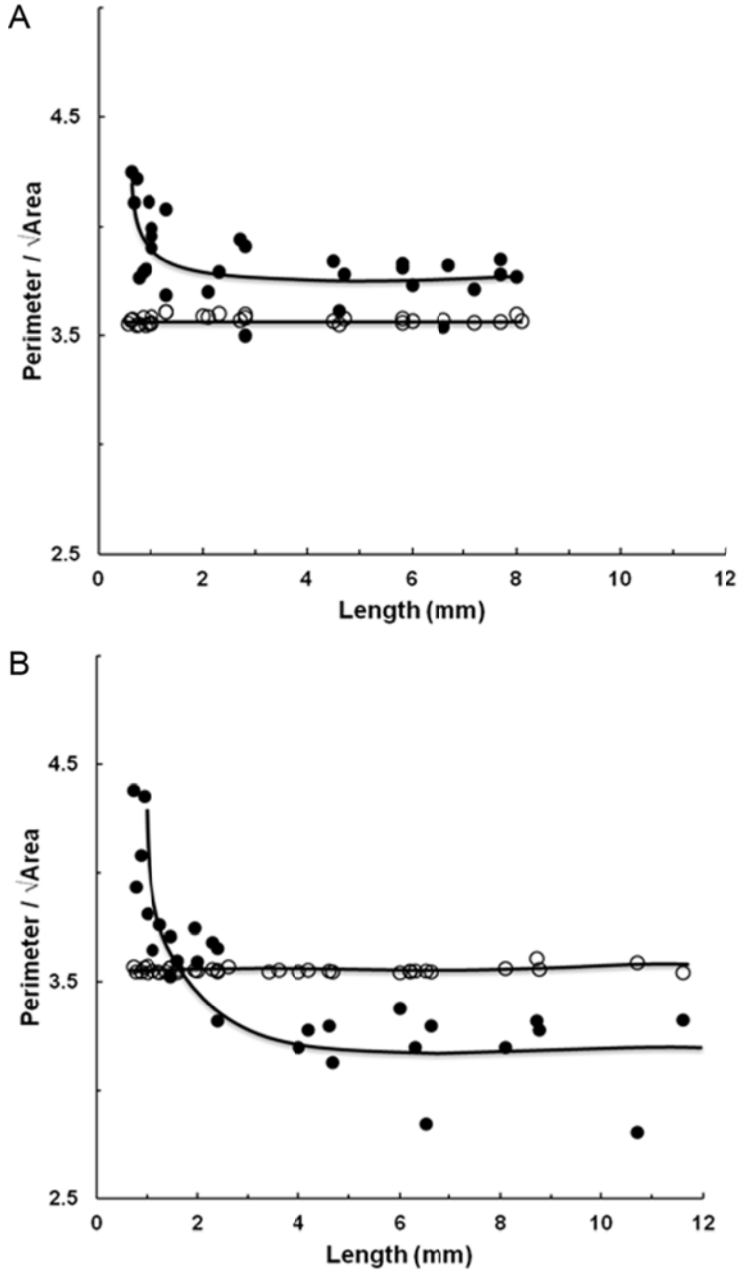

Fig. S4. P/√A values of Col-0 (A) and *tni* (B) leaves are plotted against leaf length. Open circles (○) denote the predicted P/√A values calculated from the actual leaf length and leaf width values with the assumption that the leaves are planar and elliptical in shape. The formulas for calculating the perimeter (P) and the area (A) are given in Materials and Methods. The filled circles (●) denote the actual P/√A values derived from the measured P and A values shown in Fig. 2C.

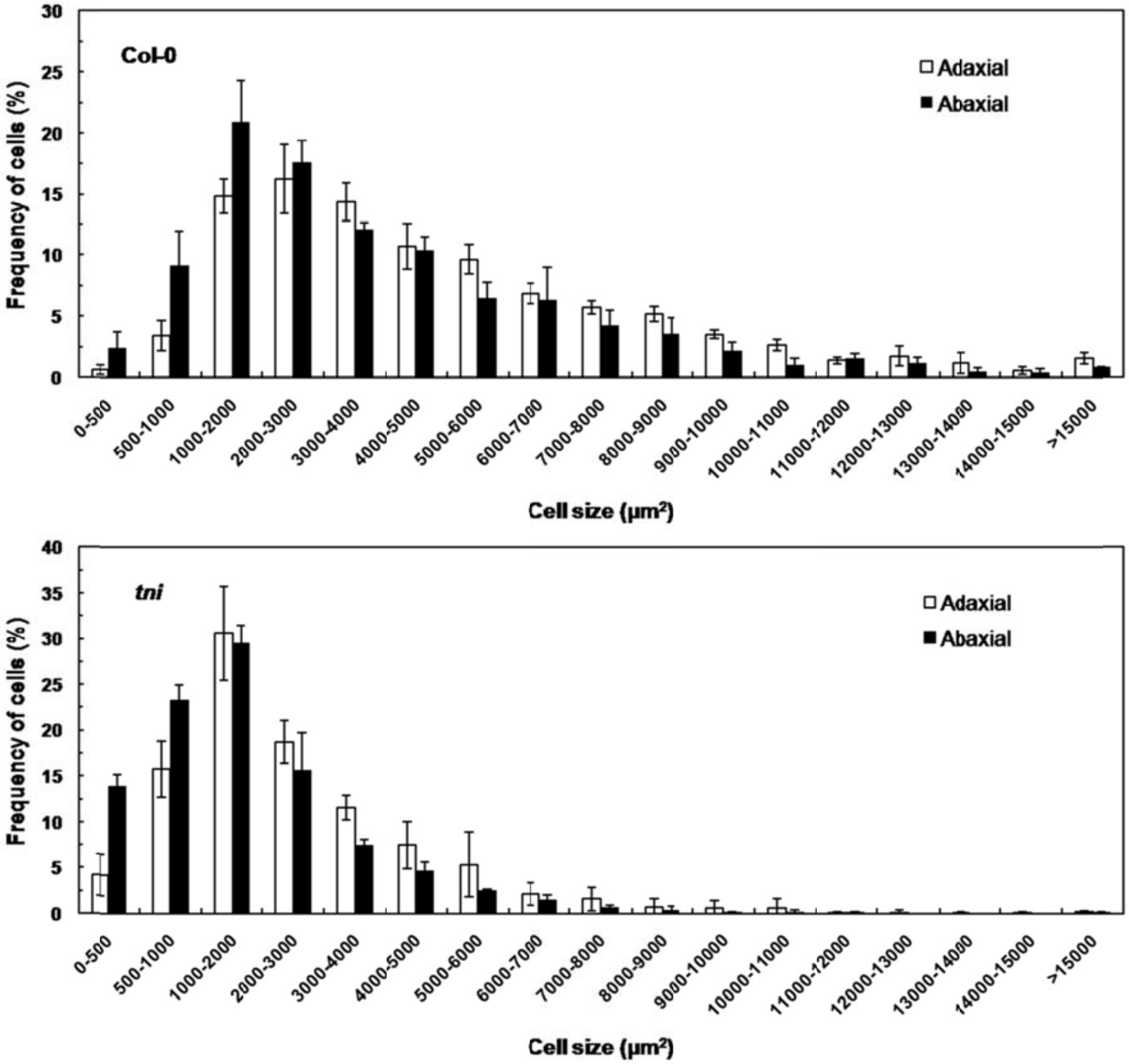

Fig. S5. Frequency distributions of epidermal cell size. Percentages of adaxial and abaxial epidermal cells of Col-0 and *tni* leaves are plotted against cell size. 1400-1700 epidermal cells in each surface (adaxial or abaxial) of 3 leaves in each genotype were measured and analyzed.

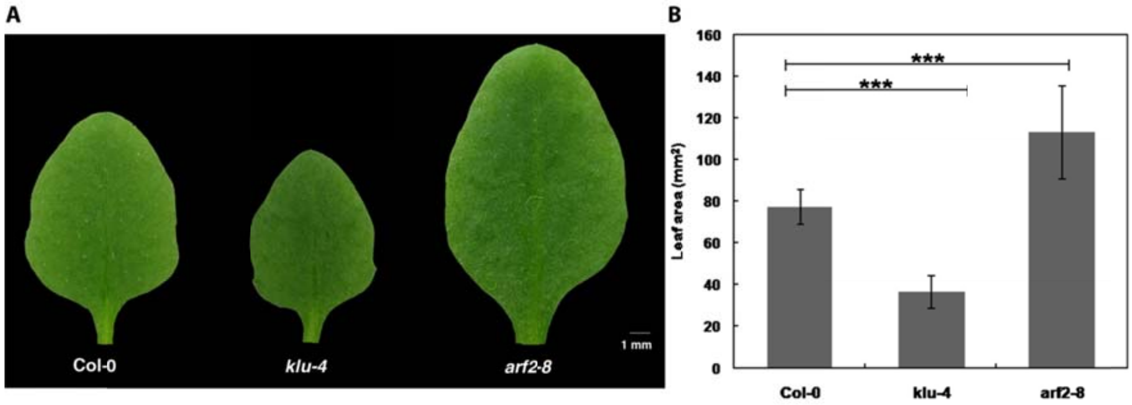

Fig. S6. Mutants with altered leaf size in *Arabidopsis*.(A) Mature leaves on the 5th node from the base of indicated genotypes.(B) Average leaf size of indicated genotypes is shown. Error bars indicate SEM. Sample numbers are 11 (Col-0), 15 (*klu-4*) and 9 (*arf2-8*) leaves. *** signifies P≤0.001. Student's T test was used.

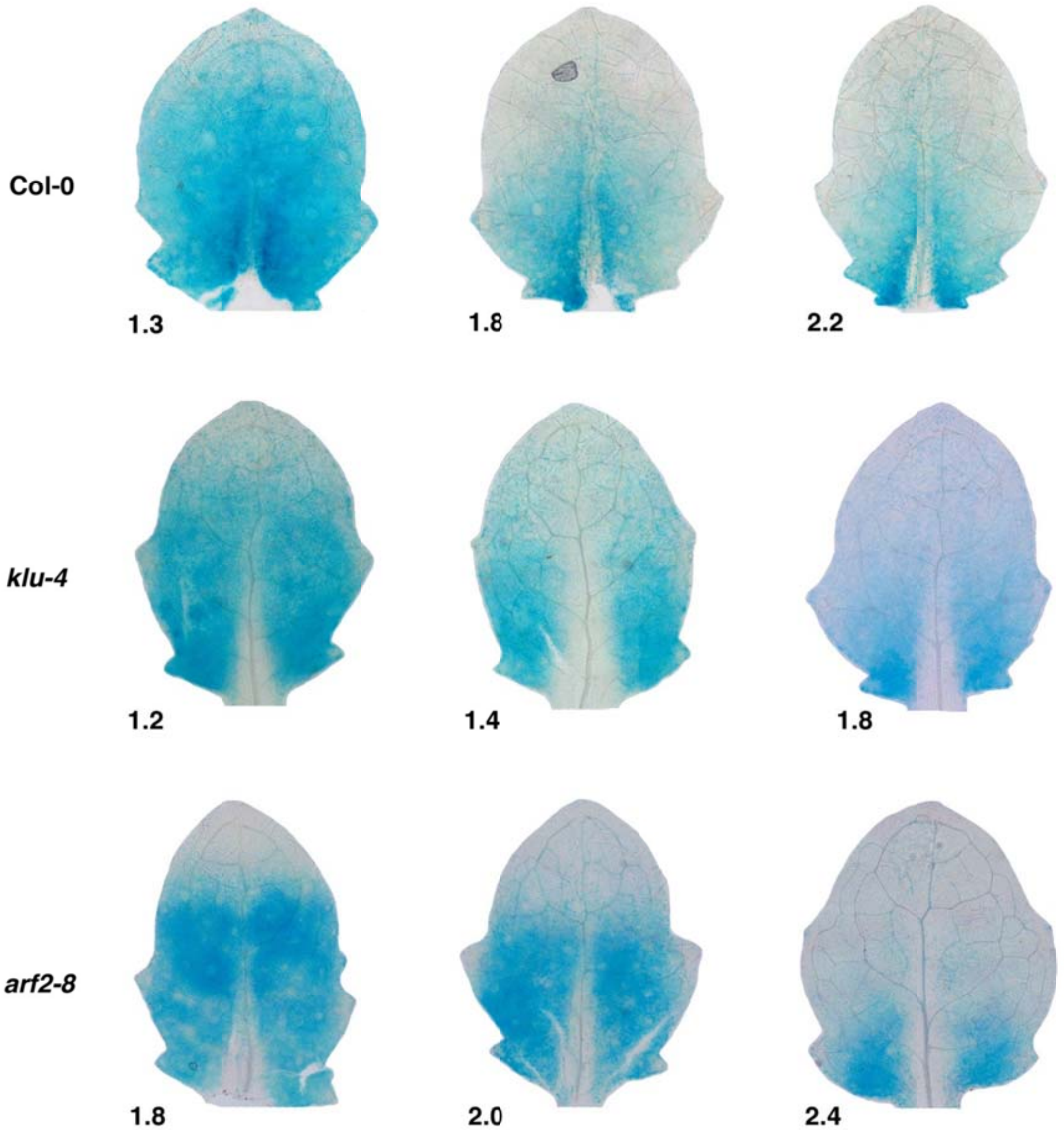

Fig. S7. Mitotic arrest zone in mutants with altered leaf size. *CYCLIN D3;2* expression is shown as GUS reporter activity (blue dots) in young leaves of indicated genotypes. Numbers denote leaf length in mm.

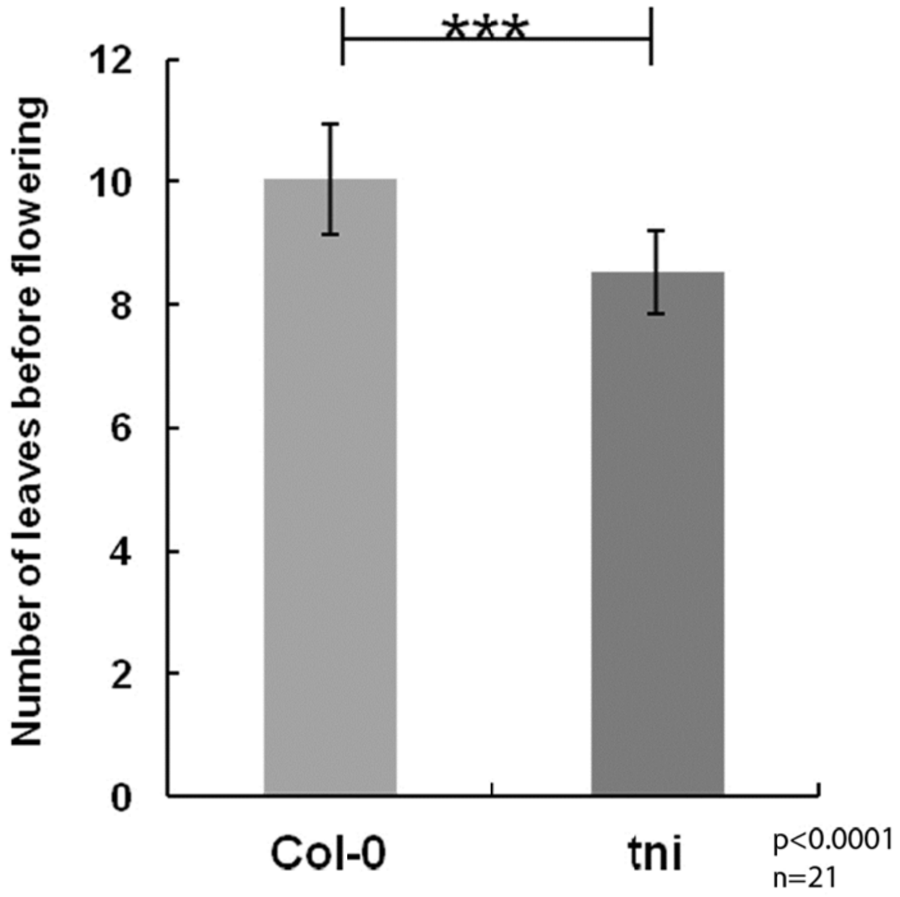

Fig. S8. Flowering time of Col-0 and *tni*. Number of rosette leaves produced by Col-0 and *tni* plants growth under long day condition (16hrs/8 hrs) is shown as a read-out of flowering time.

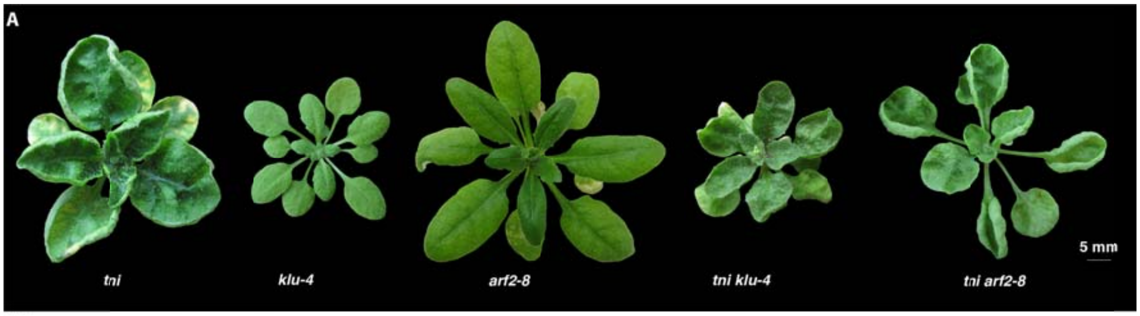

B

Genotype	Length (mm)	Width (mm)	Perimeter (mm)	Area (mm^2)	Length/ Width	SEM	Perimeter/ √Area	SEM	N
tni	14.5 ± 1.3	15.8 ± 1.4	40.6 ± 1.7	182.2 ± 32.2	0.9	0.03	3.0	0.07	10
klu-4	8.4 ± 0.8	5.6 ± 0.8	23.0 ± 2.4	36.3 ± 8.6	1.5	0.04	3.8	0.03	15
arf2-8	14.9 ± 1.4	10.0 ± 1.0	40.0 ± 3.5	111.9 ± 24.3	1.4	0.02	3.8	0.02	9
tni klu-4	8.0 ± 0.8	8.5 ± 0.9	26.8 ± 2.4	54.9 ± 10.6	0.9	0.02	3.6	0.06	14
tni klu-4 (8th leaf)	12.3 ± 1.6	14.8 ± 1.2	40.1 ± 3.4	161.2 ± 26.6	0.8	0.04	3.2	0.05	10
tni;arf2-8	15.4 ± 1.7	17.7 ± 1.4	49.7 ± 5.7	240.1 ± 46.2	0.9	0.02	3.1	0.06	7

Fig. S9. Surface curvature of *tni* leaf is independent of leaf size.(A) 1 month-old rosettes of indicated genotypes are shown.(B) Shape and size parameters of mature leaves (on 5th node from the base, unless otherwise indicated) of indicated genotypes. Mean values with SEM are shown. N indicates sample size. Note that the cup-shaped phenotype of *tni* leaves are maintained even if the lamina size is either decreased or increased in the *tni klu-4* or *tni arf2-8* backgrounds, respectively.

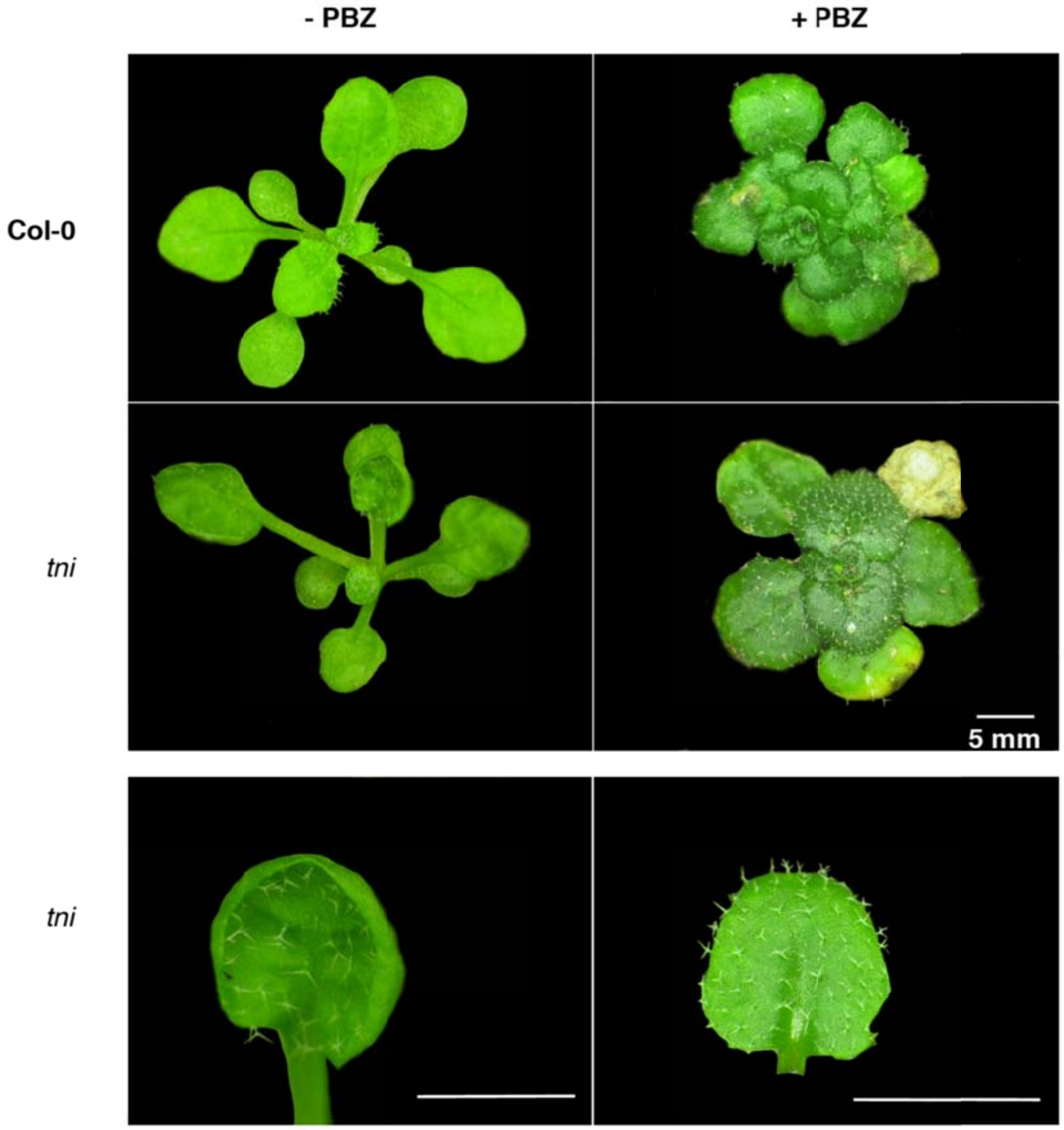

Fig. S10. Rescue of *tni* leaf phenotype by systemic application of paclobutrazol. 1-month old rosette of Col-0 and *tni* plants with (+PBZ) and without (-PBZ) the treatment with paclobutrazol (upper panel). Rescue of positive curvature in *tni* leaf upon PBZ treatment (lower panel). Scale bars, 5 mm.

A

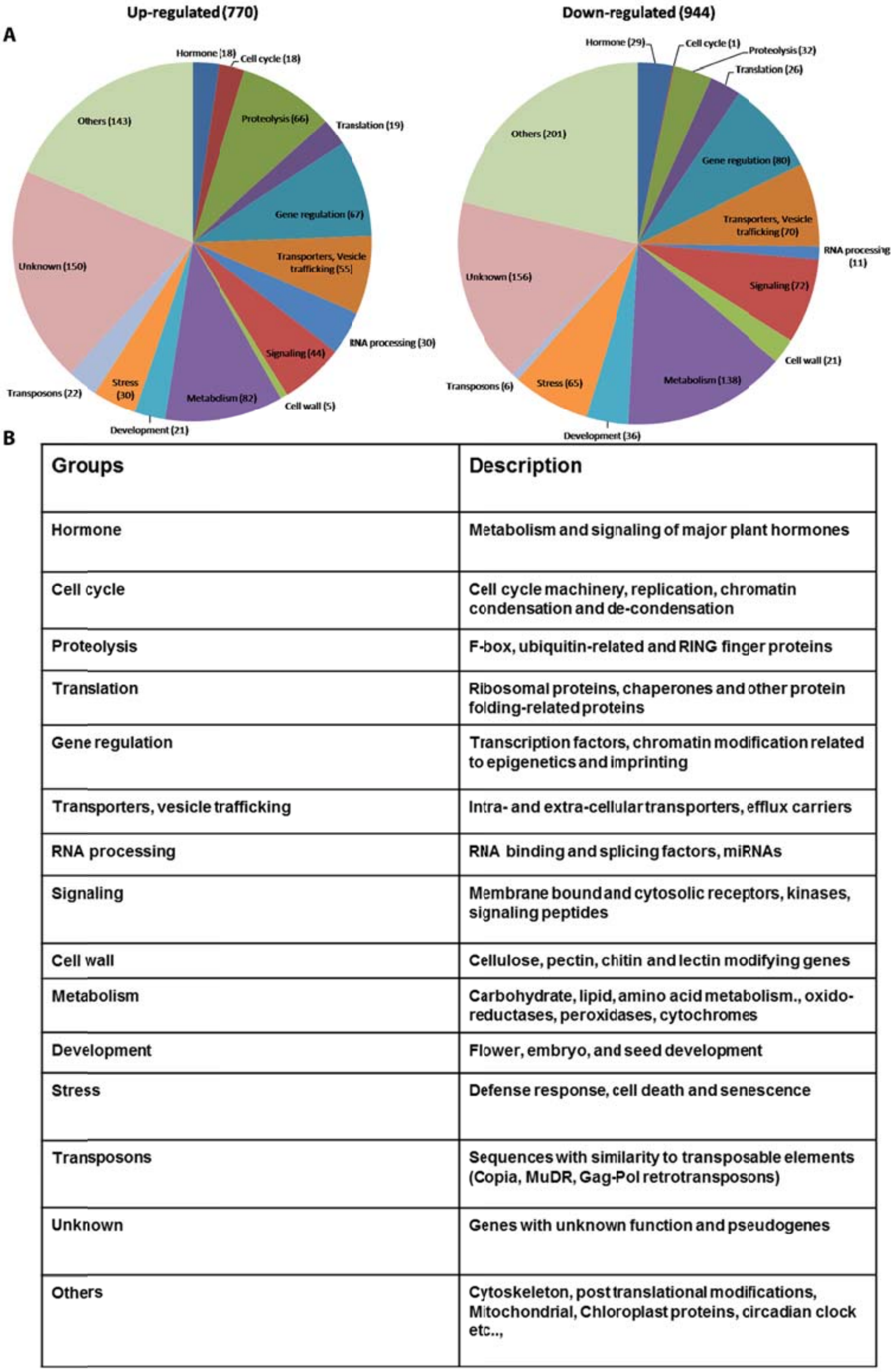

B

Groups	Description
Hormone	Metabolism and signaling of major plant hormones
Cell cycle	Cell cycle machinery, replication, chromatin condensation and de-condensation
Proteolysis	F-box, ubiquitin-related and RING finger proteins
Translation	Ribosomal proteins, chaperones and other protein folding-related proteins
Gene regulation	Transcription factors, chromatin modification related to epigenetics and imprinting
Transporters, vesicle trafficking	Intra- and extra-cellular transporters, efflux carriers
RNA processing	RNA binding and splicing factors, miRNAs
Signaling	Membrane bound and cytosolic receptors, kinases, signaling peptides
Cell wall	Cellulose, pectin, chitin and lectin modifying genes
Metabolism	Carbohydrate, lipid, amino acid metabolism., oxido-reductases, peroxidases, cytochromes
Development	Flower, embryo, and seed development
Stress	Defense response, cell death and senescence
Transposons	Sequences with similarity to transposable elements (Copia, MuDR, Gag-Pol retrotransposons)
Unknown	Genes with unknown function and pseudogenes
Others	Cytoskeleton, post translational modifications, Mitochondrial, Chloroplast proteins, circadian clock etc..,

Fig. S11. Global transcriptome analysis of *tni* leaves. (A, B) Transcripts differentially expressed in 1-3 mm long *tni* leaves on the 5th node from the base are classified into functional groups and plotted as a pie chart (A). Numbers in parentheses indicate number of genes up- or down-regulated. The functional groups are defined in (B).

Table S1. Sequences of primers used in this study.

Gene	Primer sequence
TCP2	**5'-CTCCTTCTTTAAATCCCAAACCAACC-3'**<br>**5'-GGATTCTGCCGGTGATATCAAATGG-3'**
tcp2	**5'-TAGCATCTGAATTTCATAACCAATCTCGATACAC-3'**<br>**5'- GGATTCTGCCGGTGATATCAAATGG-3'**
TCP4	**5'-ATGTCTGACGACCAATTCCATC-3'**<br>**5'-TCAATGGCGAGAAATAGAGGAA-3'**
tcp4	**5'-ATGTCTGACGACCAATTCCATC-3'**<br>**5'-TAGCATCTGAATTTCATAACCAATCTCGATACAC-3'**
TCP10	**5'-ACAAAGCAAGTGGGCAACAAAAACG-3'**<br>**5'-TAGTTTAGAGGTGTGAGTTTGGAGG-3'**
tcp10	**5'-AACTTCTGCTATCCTTTCCACCA-3'**<br>**5'-GTTACCCAACTTAATCGCCTTG-3'**
PPD1	**5'-CAAGATACCGAAACGTGGAGATGCT-3'**<br>**5'- GGTGGTGTCAAAGTAAGACTCGAG-3'**
PPD2	**5'-CGGGTCAAAAGGCGGGAAGAACAAT-3'**<br>**5'-GCCACATCCTCTCTCTCTCTCTC-3'**
35S::ICK2	**5'-CGCAAGACCCTTCCTCTATA-3'**<br>**5'-GCGGCGAGACTCTACATCTT-3'**
**TN3C6.4**	**5'-CAT TGG CAG AGG CAA CTC GTT TGC-3'**<br>**5'-GTA TAG CCC ACA CGA GCA GAT ACC-3'**
**TN3C6.7**	**5'-GAG GCC ACA CCA CTC TCT GTA CAA-3'**<br>**5'-CCT TAG CTG TAA CCA CTA TCA CAC ACT G-3'**
**TN3C7.09**	**5'-CTC AGC ACG AAA GAT TCT AGT TCA TAT GTG-3'**<br>**5'-GGC AAT GGA AAA TTA TGG AAA GGT GGG A-3'**
**TN3C7.5**	**5'-TGG AGC TCA AAT ACT TGC CCG GCA-3'**<br>**5'-CTC TTT GTC TCT TCC TCC ACC ACA G-3'**

Table S2. The p-values of the Student's t-test carried out on the data shown in Table 1.

Genotype	Length	Width	Perimeter	Area	Length/ Width	Perimeter/ √ Area
**Col-0 &*tni***	0.029	<0.0001	< 0.0001	< 0.0001	0.0001	0.0001
**Col-0 &*jawD***	0.0112	0.0029	0.0001	< 0.0001	1	0.0001
tni&tni jaw-D	0.2621	0.2605	< 0.0001	< 0.0001	0.0085	< 0.0001
**L*er*&*ppd***	0.0027	0.847	0.3188	0.099	0.0017	0.0002
ppd&jaw-D ppd+/-	0.607	0.0007	0.0053	< 0.0001	0.0001	0.0001
**Col-0 &*35S::ICK2* (het)**	<0.0001	<0.0001	< 0.0001	< 0.0001	0.0066	0.7094
tni&tni 35S::ICK2	0.0016	< 0.0001	< 0.0001	< 0.0001	0.4685	0.0539
tni&tni ga1-3	<0.0001	< 0.0001	< 0.0001	< 0.0001	0.0592	0.0017
**Col-0 (- PBZ) & Col-0 (+ PBZ)**	<0.0001	< 0.0001	< 0.0001	< 0.0001	0.012	0.1204
**Col-0(+ PBZ) &*tni*(+ PBZ)**	0.8762	0.0326	0.0071	0.1068	0.1064	0.8305
**Col-0& *kluh-4***	<0.0001	< 0.0001	< 0.0001	< 0.0001	0.0708	0.4381
**Col-0 & *arf-8***	0.0019	0.0088	0.0151	0.0085	0.1487	0.748
tni&tni kluh-4 (5th leaf)	<0.0001	< 0.0001	< 0.0001	< 0.0001	0.1619	< 0.0001
tni&tni kluh-4 (8th leaf)	0.0204	0.0456	0.1362	0.0982	0.0282	0.7273
tni&tni arf2-8	0.3624	0.0609	0.0024	0.0097	0.1028	0.7849

Table S3. Comparison of transcripts differentially expressed in *tni* leaves with those in GA3-treated leaves [GA3 study 3 (*p35S:HF-RPL18*) / untreated *p35S:HF-RPL18* rosette samples] *tcp* loss-of-function mutants [*tcp2/tcp4ko*/*TCPp::wtTCP:GFP*] and *TCP4* gain-of-function lines [*TCP4p::rTCP4:GFP* / *TCPp::wtTCP:GFP*]. Numbers indicate the number of genes up- or down-regulated in the specified genotypes.

	GA3 study		*tcp2;tcp4*		*rTCP4:GFP*	
	Up	Down	Up	Down	Up	Down
tni Up	46	73	47	35	128	196
tni Down	141	79	108	38	238	357
